# Supplementary material for: Treatment of early hypertension among persons living with HIV in Haiti: Protocol for a randomized controlled trial
Source: PLoS One. 2021 Aug 5;16(8):e0254740. doi: 10.1371/journal.pone.0254740 (PMC8341523; doi:10.1371/journal.pone.0254740)
Supplement: S1 File — (PDF) [file pone.0254740.s002.pdf]

Treatment of Early Hypertension among Persons Living with HIV in Haiti

Sponsored by:

NIH Fogarty International Center

Principle Investigator WCM:

Margaret McNairy MD MSc

Principle Investigator GHESKIO:

Jean William Pape MD

Version Number:

Version 1.0, dated March 30, 2020

Letter of Amendment #1, dated Sept 28 2020

STATEMENT OF COMPLIANCE

The study will be carried out in accordance with Good Clinical Practice (GCP) as required by the following:

- United States (US) Code of Federal Regulations (CFR) applicable to epidemiological studies (45 CFR 46);
- International Conference on Harmonisation (ICH) E6; 62 Federal Register 25691 (1997);
- National Institutes of Health (NIH) Clinical Terms of Award, as applicable;
- Weill Cornell Institutional Review Boards (IRB);
- Groupe Haitien d'Etude du Sarcome et des Infections Opportunties (GHESKIO) IRB;
- International and local requirements.

Compliance with these standards provides public assurance that the rights, safety and well-being of study subjects are protected, consistent with the principles that have their origin in the Declaration of Helsinki and The Belmont Report: Ethical Principles and Guidelines for the Protection of Human Subjects of Research of the US National Commission for the Protection of Human Subjects of Biomedical and Behavioral Research (April 18, 1979).

All key personnel (all individuals responsible for the design and conduct of this study) have completed Human Subjects Protection Training.

The study and consent forms will be approved by an institutional review board at Weill Cornell and at GHESKIO.

Protocol Summary

Title: Prehypertension in Haiti

Study Summary: We propose an unblinded pilot RCT of 250 persons living with HIV (PLWH), aged 18-65 years, who receive ART care at GHESKIO, are virally suppressed, and have pre-HTN (SBP 120-139 or DBP 80-89 mm Hg) to be randomized to **early HTN treatment** versus **standard of care** (SOC). Participants will be recruited from GHESKIO's HIV clinic. Eligible individuals will complete informed consent and be randomized to early HTN treatment vs. SOC. Participants in early HTN treatment will initiate amlodipine immediately versus those in SOC will initiate amlodipine only if they meet the HTN threshold during the study period. All participants will be followed for 12 months. The primary outcome is mean change in SBP at 12 months between study arms. Secondary outcomes are viral suppression, medication adherence, adverse events, and acceptability. We will also assess CVD risk factors and myocardial/vascular dysfunction among all participants at enrollment.

Population: We will recruit 250 PLWH from GHESKIO'S HIV Clinic. Inclusion criteria: PLWH 18-65 years of age, ART duration ≥ 1 year , stable regimen ≥ 6 months, HIV 1-RNA < 1,000 copies/mL within past 12 months, Pre-HTN (SBP 120-139 or DBP 80-89 mm Hg), no current antihypertensive treatment, receives HIV care at GHESKIO, and willing to provide consent. Exclusion criteria: pregnancy, kidney disease, diabetes, advanced illness with limited life expectancy, plans to move out of the area within the next year, and clinician determination that patient is unstable on ART.

Number of Sites: Single site: GHESKIO Center Port au Prince, Haiti

Study Duration: 2 years

Subject Duration: 12 months

Primary Aim: To assess the feasibility of initiating antihypertensive treatment among PLWH with pre-HTN through a pilot randomized controlled trial comparing early HTN treatment to SOC. Feasibility outcomes include enrollment and retention data, proportion who initiate amlodipine, and incremental change in SBP at 12 months between arms. The pilot trial has ≥80% power to detect differences in change in SBP ≥ 4 mm Hg.

Secondary Aims: To also examine the following outcomes and measures:

a. Viral suppression compared between study arms at 12 months (defined as HIV-1 RNA < 1000 copies/ml)
b. HIV medication adherence compared between study arms at 6 and 12 months (defined as >90% adherence using 3-day pill recalls)
c. Adverse events compared between study arms at 12 months (defined as Grade III-V adverse events including hypotension, peripheral edema, and other symptoms which may be related to amlodipine)
d. Acceptability assessed through in-depth interviews with a subset of participants and providers

e. CVD risk profile measured among all participants at enrollment including obesity, dyslipidemia, diabetes, smoking, physical inactivity, poor diet, and 10-year CVD risk (only among participants $\geq$ 40 years)
f. Pre-existing myocardial and vascular dysfunction measured among all participants at enrollment using ECG, echocardiography and vascular ultrasound

Study endpoints and analysis: The primary endpoint is the difference in mean change in SBP (mm Hg) between study arms at 12 months. Secondary outcomes include viral suppression and medication adherence. We will also measure CVD risk factors among participants. We will compare the mean difference in change in SBP among participants using a linear mixed-effects model (LMM)accounting for repeated measures and correlations within subjects. We will assess incidence of HTN using Kaplan Meier methods. Dichotomized outcomes such as HTN will be analyzed via generalized linear mixed-effects model (GLMM) at all time pints and via Fisher’s exact test and logistic regression at 12 months. Viral suppression, medication adherence, and CVD risk factors will be compared between study arms using Fisher’s exact test. Longitudinal data such as BMI and adherence will be analyzed using LMM and GLMM.

1 KEY ROLES

Principal Investigator (WCM):

Margaret McNairy, MD, MSc
Associate Professor of Medicine
Center for Global Health
Weill Cornell Medicine
402 East 67th Street
New York, NY 10065
Work: 646-962-8140
Email: mam9365@med.cornell.edu

Principal Investigator (GHESKIO):

Jean William Pape, MD
Professor of Medicine
Director, GHESKIO Centres
33 Boulevard Harry Truman
Port-au-Prince, Haiti
Work: 509 3702 9366
Email: jwpape@gheskio.org

Co-Investigators:

Samuel Pierre, MD
Senior Research Physician
GHESKIO Centres
33 Boulevard Harry Truman
Port-au-Prince, Haiti

Vanessa Rouzier, MD
Chief of Pediatrics
GHESKIO Centres
33 Boulevard Harry Truma
Port-au-Prince, Haiti

Myung Hee Lee, PhD
Assistant Professor, Center for Global Health
Weill Cornell Medicine
402 East 67th Street
New York, NY 10065
Email: myl2003@med.cornell.edu

Lily Yan, MD, MSc
Instructor of Medicine
Center for Global Health
Weill Cornell Medicine
402 East 67th Street
New York, NY 10065
Email: liy9032@med.cornell.edu

Consultants:

Suzanne Oparil
Professor of Medicine
University of Alabama-Birmingham Medical School
170 University Blvd
Birmingham, AL 35233

2 BACKGROUND INFORMATION AND SCIENTIFIC RATIONALE

2.1 Background Information

Figure 1: Map of Haiti

FIGURE REDACTED

Haiti is located on the western third of the island of Hispaniola in the Caribbean Sea and is the poorest country in the Western Hemisphere (Figure 1). Haiti ranks 163 out of 188 countries in the UN Human Development Index, which measures economic, education, and health factors.[1] Haiti has the highest HIV prevalence in the hemisphere (2%); currently an estimated 140,000 adults are living with HIV, with two-thirds of cases in Port-au-Prince.[2-4] Over the last decade, **CVD surpassed HIV as the leading cause of morbidity and mortality in Haiti**.[5-8] In 2016, the Global Burden of Disease Project estimated the most common cause of death among adults was CVD (27.2% of deaths) in comparison to HIV (5.6% of deaths).[8] Recent cross-sectional data suggest that 20% of Port-au-Prince residents have HTN (29% age-standardized).[9 10] Haiti has one of the **highest stroke mortality rates** in the Latin America/Caribbean region, and more than twice that of the neighboring Dominican Republic.[11] One of the strongest stroke risk factors is HTN.

Cornell-GHESKIO has a **37-year track record** of NIH-supported research in Haiti which has informed WHO and national policies on prevention and treatment guidelines for HIV and associated comorbidities.[12-20] GHESKIO was founded in 1982 with research evolving from early epidemiologic studies to a major NIH-supported Clinical Trials Unit (CTU) in 2006. This work contributed to the drop in HIV prevalence in the adult population in Haiti, from 6% in 1993 to 2% in 2016, with 60% of those infected on ART.[12] Since 2012, the CTU has **conducted over 20 clinical trials with 98% retention**. GHESKIO is trusted by the Haitian community, the Haitian Government, and international partners.[21] GHESKIO has never closed its clinics in its tenure, even during the 2010 earthquake, cholera epidemic, and periods of political instability.

In 2015, GHESKIO established the Non-communicable Disease (**NCD) Research Unit**, led by the PI of this proposal, to evaluate CVD among HIV patients and the general population. With NIH support (D42TW0009606-S1), we conducted the first population-based survey of HTN in the slums of Port-au-Prince and found that 12% of 18-30 year-olds had HTN not accompanied by risk factors such as obesity, smoking, and diabetes; this prevalence is ~3 fold higher than similarly aged Black Americans.[9 10] In 2018, we received funding (R01 HL143788 2018-2023; McNairy, PI) to establish the **country's first longitudinal cohort to evaluate the epidemiology of HTN** including CVD risk factors (e.g. diabetes, kidney disease, HIV, diet, inflammation), diseases (stroke, myocardial infarction, heart failure, and cardiac death), and poverty-related social determinants. This study dramatically increased our capacity to conduct CVD trials through assessment of CVD risk factors, training in ECG and echocardiography, validation of community-based BP measurement, and formation of a CVD adjudication committee. In 2019, the PI of this proposal led a collaboration with the Ministry of Health and international experts to develop Haiti's first **national primary care HTN guidelines** based on WHO recommended algorithms.[22 23] **Amlodipine,** was selected for its safety, the lack of laboratory monitoring, and its effectiveness in Blacks.[24-26] We have also received funding (RESOLVE to SAVE LIVES, PI McNairy) for HTN medications and support for guideline implementation.[27]

<u>**HTN in PLWH is associated with mortality**</u>: Cornell-GHESKIO's CVD research began with the evaluation of CVD outcomes among PLWH who had been followed for 10 years (the *GHESKIO ART Cohort*).[28] This cohort includes 816 HIV-infected adults who started ART in 2005-2008 when ART first became widely available in Haiti. At ART initiation, their median age was 39 years (IQR 33-46), 58% were female, and their median CD4 count was 281 cells/mm^3 (IQR 250-311). At ART initiation, 5.3% had HTN. In multivariable analysis, HTN at ART initiation independently predicted mortality during 10 years of follow-up (HR 2.47 [95% CI 1.10-5.57] after adjusting for age, sex, and CD4 count at ART start). Figure 2 shows Kaplan Meier survival curves of those with normal BP and those with HTN at ART initiation. One-third of the deaths among those with HTN were from stroke. Over the median follow-up of 7.3 years, HTN prevalence increased to 43% (HTN incidence 34/1000 person-years). HTN prevalence did not differ by gender and was 18% in patients <40 years, 37% in 40-59 years, and 60% in ≥60 years. Incident HTN was strongly predictive of death.

Figure 2: Survival of HIV-infected adults with and without HTN at ART initiation

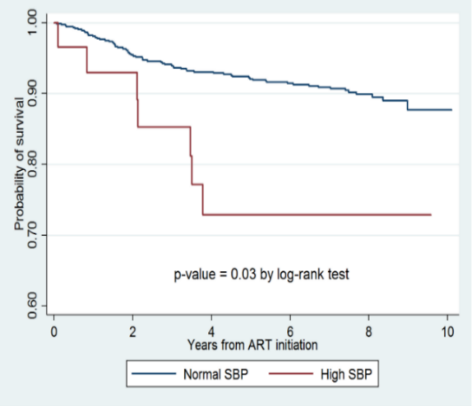

We analyzed a more recent cohort of 762 PLWH who initiated ART between 2013-2015 at GHESKIO and found HTN prevalence at time of ART initiation doubled to 12% and HTN incidence tripled to 108/1000 person-years (Table 1). Of note, 38% of PLWH with pre-HTN at time of ART start developed incident HTN within 18 months. We postulate that the GHESKIO ART Cohort had a higher number of persons with AIDS at ART start as compared to this later cohort.

Table 1: PLWH who initiated ART 2013-2015 (N=762)

HTN prevalence	12%
Pre-HTN prevalence	22%
Proportion of pre-HTN PLWH who developed HTN in 18 months	38%

Pre-HTN is associated with increased CVD risk in PLWH as compared to adults without HIV: A study from the US quantified the increased CVD risk among PLWH with pre-HTN as compared to pre-HTN adults without HIV. PLWH with pre-HTN had increased risk of myocardial infarction (adjusted HR 1.8 [95% CI 1.22-2.68]).[29] Our preliminary data show that 27% of PLWH with pre-HTN compared to 7% of HIV-uninfected adults with pre-HTN had elevated 10-year CVD risk, a >3-fold higher prevalence (Figure 3). We used the AHA Pooled Cohort Risk Equations to estimate 10-year CVD risk among adults 40-79 years.[30] Elevated 10-year risk is defined as a predicted risk of ≥7.5% or having diabetes. We included 192 PLWH (median age 45, 72% female, median duration of ART 3 years) and 153 adults without HIV (median age 41 years, 61% female). The relative contributions of specific risk factors explaining this increase among PLWH need further investigation.

Figure 3:
Prevalence of Elevated 10-year CVD risk among HIV-infected and uninfected adults

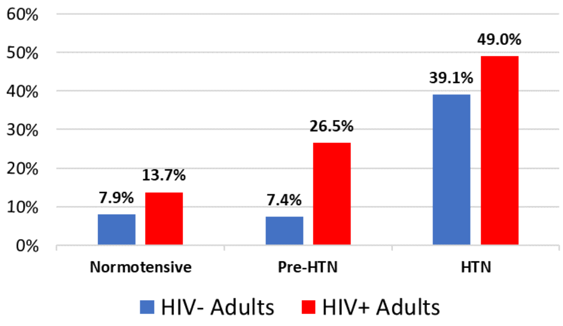

2.2 Scientific Rationale

HIV-associated CVD has tripled over the past two decades globally and HTN is a major driver of CVD among PLWH.[29 31-35] Around the globe, >35% of PLWH have HTN, and >50% of PLWH >50 years of age have HTN.[33] In addition to traditional CVD risk factors, the pathophysiologic mechanisms leading to HTN among PLWH may include virologic and treatment-related factors influencing inflammation, immune mediation, lipodystrophy and the renin-angiotensin-aldosterone system.[36-40]

Table 2: Summary of WHO and Haitian HTN definitions and guidelines

Category	Definition	Threshold for initiation of antihypertension treatment
Normal	SBP < 120 and DBP < 80	
Pre-HTN	SBP 120-139 or DBP 80-89	Diabetes & Chronic Kidney Disease (at SBP ≥ 130 or DBP ≥ 90)
HTN	SBP ≥ 140 or DBP ≥ 90	General adults including PLWH

The definition of pre-HTN and HTN according to WHO, international and Haitian Ministry of Health guidelines are listed in Table 2.[22 23 41] The relationship between SBP >115 mm Hg and CVD events is linear, with multiple epidemiological studies reporting increased risk of CVD, renal disease, and all-cause mortality among adults with pre-HTN.[42-46] Untreated pre-HTN is a progressive condition of ongoing arteriolar hypertrophy, endothelial dysfunction, and stimulation of the renin-angiotensin system which lead to further HTN.[47-49] Landmark trials and meta-analyses have shown that 1) treatment of pre-HTN in patients with diabetes, chronic kidney disease, and nonobstructive coronary disease decreases CVD events and disease progression,[50-54] and 2) use of antihypertensive treatment among individuals with pre-HTN can prevent progression to HTN.[47] These data led to revised guidelines recommending antihypertensive treatment at lower BP thresholds among individuals with these diseases.[22 41 55] In 2017, the US guidelines went further to recommend antihypertensive treatment for high-risk adults with SBP ≥130 or DBP ≥80 mm Hg, but HIV is not currently included as a high-risk category.[55]

HIV is associated with equal, if not greater, CVD risk as diabetes;[56] yet, the WHO still recommends PLWH initiate antihypertensive treatment at the same BP threshold as adults without HIV. Data are needed to clarify if initiation of antihypertensive treatment among PLWH with pre-HTN can prevent progression to HTN, and ultimately CVD-related mortality. The proposed pilot trial is the first step to explore the feasibility, benefits, and risks of initiating antihypertensive treatment among PLWH with pre-HTN in a well-established HIV program in Haiti, one of the poorest countries in the world. Knowledge gaps include: Is it feasible to initiate and integrate antihypertensive treatment into HIV care among relatively healthy PLWH with pre-HTN? Could initiating antihypertensive treatment worsen HIV outcomes? What is the BP reduction using amlodipine (which is Haiti's first-line antihypertensive medication) among PLWH with pre-HTN? Data from this pilot trial will inform the design of a future multicenter trial powered for incident CVD events. Our **goal is to change guidelines** to lower the BP threshold for initiation of antihypertensive medications among PLWH, should earlier HTN treatment prevent CVD.

3 POTENTIAL RISKS AND BENEFITS

3.1 Potential Risks

The risks of this study include loss of confidentiality of study participation, social harms, discomfort, and phlebotomy, and adverse events of medications. GHESKIO has experience conducting research studies and has produced teaching materials in Creole for the risks involved in these studies during the informed consent process. Further, the occurrence of these adverse events will be closely monitored by the study team and reported to the DSMB and IRB.

A. Loss of Confidentiality: There is a potential loss of confidentiality of information provided during the study including one's HIV status or diagnosis of hypertension through participation in the study.

B. Social Harms: Participants may experience social harms including loss of privacy, or social stigma from perception of being involved in a study related to HIV and cardiovascular disease risk factors, such as hypertension. HIV-infected adults are a particularly stigmatized population at heightened risk of social harms.

C. Discomfort: Participants will be asked to complete a questionnaire at study enrollment and at the 12 months study visit. The questionnaire includes topics that may be sensitive such as family history and health behaviors including smoking, alcohol, physical activity, and diet.

D. Phlebotomy: There is a slight risk of physical discomfort (i.e., slight bruising or tenderness at the phlebotomy site) to participants associated with collection of blood sample for hematology, chemistry, and HIV viral load. Approximately 15 mL of venous blood will be collected by phlebotomy at study enrollment for these laboratory measures. Approximately 5 mL of venous blood will be collected by phlebotomy for HIV viral load testing at the 12-month study visit.

E. Adverse events: Amlodipine is recommended by the WHO as first-line medication for elevated blood pressure and has a safe side effect profile.[22] The most common side effects of amlodipine are dizziness and or lightheadedness and peripheral edema. We will monitor adverse events using DAIDS criteria for hypotension, peripheral edema and other side effects reported by participants that are related to amlodipine. In other studies, a dose of amlodipine 10mg daily is associated with ~ 5-6 mm Hg reduction in SBP over 1 month.[57,58] In one clinical trial (CAMELOT), which evaluates the use of amlodipine 10 mg daily among normotensive adults with existing cardiovascular disease, 3.3% of participants reported symptoms of lightheadedness and dizziness for which amlodipine was discontinued and 5% discontinued amlodipine due to peripheral edema.[58] We anticipate a similar rate of side effects and adverse events in this study.

Protection against risks:

The following measures will minimize study risks:

A. Loss of Confidentiality: Each participant will be assigned a study identification number consisting of a unique code unrelated to any patient identifying information, as per Good Clinical Practice guidelines. Any identifiers linked to the participant will be secured and only available to staff at

the GHESKIO Research Center, to ensure subject confidentiality. Only authorized professionals collaborating with the principal investigator will have access to the information. All samples and subject data will be coded at the GHESKIO Research Center with the study identification number. All source documents will be kept in locked file cabinet. GHESKIO computers and servers are protected by firewall protection and are only accessible with a password. Study staff will be trained in Good Clinical Practice regarding maintaining confidentiality. Participants will be asked to report any breeches in confidentiality, and these will be reported to the DSMB.

B. Social Harms: Research staff are trained on how to respond to any social harm reported by a participant. Staff performance will be monitored by the study director, Dr. Pierre during quality assurance assessments to ensure minimization of social harms. Doctors Pape, McNairy, and Pierre will be available to assist the study staff in responding to any event that requires further management. We routinely educate all patients in the GHESKIO clinic on the importance of confidentiality and not sharing information with other patients. Participants will be encouraged to report any adverse event or social harms including loss of confidentiality and stigma to study staff. These will be documented in source documents and reported to the study team on standardized adverse event forms.

C. Discomfort: As part of the informed consent process, all potential participants will be instructed that they do not have to disclose personal information that they are uncomfortable sharing and that they can withdraw from the study at any time.

D. Phlebotomy: Participants will be informed of the possible risks of slight bruising or tenderness at the site of the blood draw. They will be encouraged to call or visit the study site if symptoms persist or worsen.

E. Adverse events: Participants will be informed of the possible side effects of amlodipine including hypotension and peripheral edema. We will grade the signs and symptoms of hypotension and peripheral edema, or any other reported side effect, using existing DAIDS criteria(11) and report Grade III-V events to the DSMB and the GHESKIO and Weill Cornell IRB. Study staff will screen for drug-related adverse events at every study visit. Participants who report adverse events will discuss them with their clinical (non-research) physicians and decide together if amlodipine should be discontinued. All clinical care of any adverse event will be provided free of charge by GHESKIO.

3.2 Known Potential Benefits

The proposed study has the potential to change the paradigm of antihypertensive treatment in PLWH, both in the US and abroad. If the study finds earlier initiation of antihypertensive treatment is a form of CVD prevention, then we can reduce CVD events and related mortality in PLWH.

Participants in the proposed study will also benefit from having free screening for hypertension and free medications. Moreover, participants who are diagnosed with a cardiovascular risk factor, abnormal laboratory value (e.g. elevated hemoglobin A1C), and/or other diseases will be referred to ongoing clinical care at GHESKIO or a GHESKIO-affiliated hospital. GHESKIO is affiliated with 4 hospitals in Port-au-Prince including Hospital Bernard Mevs, Hospital du Canape, Hospital Saint-Damien, and Hospital Saint Francois de Sales. **Receipt of clinical care is not dependent on study**

participation. The GHESKIO NCD Research Unit follows national guidelines for the management of chronic diseases including hypertension, diabetes, kidney disease, among others. All WHO Essential Medications are available at the clinic including aspirin, amlodipine, hydrochlorothiazide, hydralazine, bisoprolol, enalapril, digoxin, metformin, insulin, among others. The GHESKIO affiliated hospitals provide free care to patients who cannot pay.

If the intervention study arm, early HTN treatment, is found to be effective in the primary study outcome of reduced mean SBP $\geq$ 4 mm Hg between study arms, we will continue amlodipine in participants randomized to the intervention arm and offer amlodipine to all participants in the standard of care study arm.

4 STUDY PROTOCOL

4.1 Study Objective

We propose a pilot randomized controlled trial to evaluate antihypertensive treatment among PLWH with pre-HTN at GHESKIO. This study will provide critical data on the feasibility, benefits and risks of antihypertensive treatment for CVD prevention among PLWH with pre-HTN that will inform a future definitive trial powered for incident CVD events. For the pilot study, we will enroll 250 PLWH (18-65 years of age) who have been on ART for ≥ 1 years with viral suppression within past 12 months and SBP 120-139 or DBP 80-89 mm Hg and no current antihypertensive treatment; randomize them to “early HTN treatment” or the current standard of care (SOC); and follow them for 12 months. Amlodipine is the recommended first-line anti-hypertensive medication according to Haiti’s new primary care HTN guidelines. Participants in the early HTN treatment arm will initiate amlodipine 5 mg immediately, increasing to 10 mg if SBP >130 mm Hg after 1 month. Participants in the SOC arm will initiate amlodipine only if they develop HTN (SBP ≥140 or DBP ≥90 mm Hg). We hypothesize that early HTN treatment will not impact viral suppression and be feasible and safe, with a reduction in BP at 12 months compared to SOC. The primary aim is to assess the feasibility of initiating antihypertensive treatment among PLWH with pre-HTN through a pilot randomized controlled trial comparing early HTN treatment to SOC. Feasibility outcomes include enrollment and retention data, proportion who initiate amlodipine, and incremental change in SBP at 12 months between arms. The secondary aim includes analyzing the following measures: viral suppression, HIV medication adherence, adverse events, acceptability, CVD risk profile, and pre-existing myocardial and vascular dysfunction.

4.2 Study Site

The trial will be conducted at the GHEKSIO Centers in Port au Prince, Haiti.

4.3 Study Population

The study population will include 250 PLWH from GHESKIO’s HIV clinic. Inclusion and exclusion criteria are in Table 3. We included younger PLWH given the early onset of HTN in Haiti and limited the upper age to 65 years given the low incidence of HIV above this age. We exclude PLWH with kidney disease (<60mL/min/1.73m^2 using CKD-Epi equation) because Haiti’s guidelines recommend furosemide as first-line treatment for this group. We exclude diabetics (defined as fasting glucose ≥126 mg/dL or non-fasting glucose ≥200 mg/dL, or taking diabetes medications)[140, 141] because Haiti’s guidelines recommend initiation of antihypertensive medications when SBP ≥ 130 mmHg or DBP ≥ 80 mmHg. Individuals on a protease inhibitor with ritonavir will be excluded as ritonavir can increase levels of amlodipine. Women who become pregnant will be censored at time of pregnancy.

Table 3. Inclusion and exclusion criteria for the trial

Inclusion criteria	Exclusion criteria
• *PLWH 18-65 year of age*<br>• *ART duration ≥ 1 year, stable regimen ≥ 6 months*<br>• *HIV 1-RNA < 1,000 copies/mL within past 12 months*<br>• *Pre-HTN (SBP 120-139 or DBP 80-89 mm Hg)*<br>• *No current antihypertensive treatment*<br>• *Receives HIV care at GHESKIO*<br>• *Willing to provide consent*	• *Pregnancy*<br>• *Kidney disease or diabetes*<br>• *Protease inhibitor/ritonavir*<br>• *Advanced illness with limited life expectancy*<br>• *Plans to move out of the area within the next year*<br>• *Clinician determination that patient is unstable on ART*

4.4 Recruitment and Informed Consent

Screening and recruitment will follow GHESKIO Clinical Trials Unit procedures that have been highly successful in NIH trials. We will identify and invite potential participants both 1) in-person at GHESKIO's HIV clinics using real time BP measurements and 2) from GHESKIO's electronic medical record (EMR) using BP measurements in the past 12 months, age, ART duration, viral suppression, and no current antihypertensive treatment. PLWH meeting these eligibility criteria will be tagged in the EMR as participants for recruitment and a list will be given to the study team.

The research nurse will approach patients in the HIV clinics, or call PLWH on the list to introduce the study and invite interested individuals for informed consent and then a screening visit. After confirming basic eligibility criteria, the research nurse will initiate informed consent procedures among eligible participants. The informed consent process consists of two education sessions, followed by assessment of understanding and written informed consent.

After informed consent, participants will be assigned a unique study ID number and undergo a screening visit with review of eligibility criteria and screening labs. Pre-HTN is defined as ≥2 BP measurements of SBP 120-139 mm Hg or DBP 80-89 mm Hg on separate days. All BP measurements will be done according to AHA/WHO guidelines.[22 55] Screening labs will be collected (urine or serum pregnancy test, fasting or random glucose, and creatinine) to evaluate if the patient is pregnant, has diabetes, or kidney disease. We will document the number screened and reasons for study exclusion.

In 2018, 18,872 PLWH on ART received care at GHESKIO with mean duration of ART of 5.6 years, 76% on first-line regimens, 86% virally suppressed, and median age of 35 years. We estimate 3,819 PLWH at GHESKIO satisfy the eligibility criteria. Based on our past enrollment experience, we are confident 250 participants can be enrolled from this sampling frame in 6 months.

4.5 Study Enrollment

Participants that meet study eligibility criteria will proceed to study enrollment visit (~90 min). Participants will be randomized to early HTN treatment vs. SOC in a 1:1 ratio using a computer-generated random assignment. The research nurse will collect demographic and clinical data, administer a baseline questionnaire, measure unobserved BP, and perform an ECG. The questionnaire includes a CVD medical history, family history, medications, CVD health behaviors (smoking, alcohol, physical activity, and diet) using validated questions that have been used at GHESKIO and are comparable to those used in US cohorts. Enrollment labs (~15ml of venous blood) including HgbA1c, total cholesterol, HDL, CD4 and HIV viral load (unless available from EMR in the past 6 months) will be collected. Samples of serum and plasma will be stored for future studies, with documented consent from participants. The study physician will interpret the ECG and perform an echocardiogram and vascular ultrasound.

Study drug will be dispensed to participants randomized to the early treatment arm with appropriate education and counseling on adherence and potential side effects. Amlodipine will be started at 5mg at

enrollment and increased to 10mg if SBP >130 mm Hg after 1 month for participants in the early treatment arm. Locator and contact information will be verified and follow up appointment given.

4.6 Follow-up study visits and retention efforts

After enrollment and randomization assignment (including amlodipine initiation), participants will have follow-up visits either at GHESKIO (months 0.5-1, 3, 6, 9, 12) or in the community / home with CHWs (months 2, 5, 8) (table 4). All visits will include BP measurement, lifestyle counseling, adherence encouragement, and assessment of adverse events. The GHESKIO visits will also include a physical exam. Participants in the early intervention arm will receive monthly amlodipine refills. Participants in the SOC arm will initiate amlodipine only if they develop hypertension (SBP ≥140 or DBP ≥90 mm Hg).

At the 12-month study visit, participants will also complete a brief questionnaire about changes in health behaviors and have viral load measured. Participants may return to the HIV clinic at any time for symptoms, questions, or other concerns, and a BP measurement will be taken at each of these encounters. Adverse events will be assessed at each visit. Medical record abstraction will be done by research staff for any hospitalization or death among a participant.

To encourage retention and minimize missing data, GHESKIO staff will call participants 1-2 days prior to each study visit and will also call and track participants who miss a scheduled study visit. Since 2012, GHESKIO has recruited HIV-infected adults for 16 HIV trials with 98% retention.

4.7 Study Measures and Schedule

Description of study measures and their schedules are listed in table 4.

Table 4: Study Measures and Schedule

**Required forms:**	**SCREEN**	**ENROLL**	**M1**	**M2**	**M3**	**M5**	**M6**	**M8**	**M9**	**M12**
**Screening Eligibility Form**: inclusion criteria, exclusion criteria	X									
**Screening Labs:** urine/serum pregnancy test, fasting or random glucose, Creatinine	X									
**Individual Characteristics**										
**Locator information:** address and cell phone, friend/family member's cell phone		X	X	X	X	X	X	X	X	X
**Sociodemographics**: age, sex		X								
**Socioeconomic factors:** income, education, occupation, marital status, children.		X								
**Medical History:** history of CVD risk factors, CVD, HIV, tuberculosis		X								
**Family History:** stroke or MI in parents and 4 oldest siblings		X								
**Health behaviors:** current cigarette smoking,[90] alcohol[160-163], physical activity [164]		X								X
**Multidimensional Poverty**: child death, educational attainment, household construction materials, possessions		X								
**Clinical Measures**										
**Physiologic**: height, weight		X								

**Blood pressure** in community		X		X		X		X		
**Blood pressure** at clinic		X	X		X		X		X	X
**ECG**		X								
**Echocardiography and vascular ultrasound**		X								
**Enrollment Labs**: HgbA1c, total cholesterol, HDL, CD4, VL		X								
**12M Labs:** VL										X
**Medical record abstraction:** diagnoses codes (ICD-9), laboratory measures, diagnostic imaging, and cause of death among participants who receive clinical care from the GHESKIO NCD clinic, a GHESKIO-affiliated hospital, or other health facility			X	X	X	X	X	X	X	X
**Clinical Management**										
**Medication Adherence**		X	X	X	X	X	X	X	X	X
**Medical record abstraction** for development of HTN after enrollment			X	X	X	X	X	X	X	X
**Medical record abstraction** for clinical management of CVD risk factors: smoking, HLD, DM		X	X	X	X	X	X	X	X	X
**Intervention related**										
Amlodipine initiation form		X								
In depth interviews about feasibility, acceptability										X
Adverse Events			X	X	X	X	X	X	X	X

<u>BP measurement</u>: All BP measurements will follow AHA/WHO guidelines using semi-automated electronic cuffs (OMRON 742-5 series at home and HEM907 in clinic) of 3 BPs separated by $\geq$1 minute with the individual seated at rest.[22 55] The average of the 3 BPs is the BP for the visit.

<u>HIV services for all participants:</u> Provision of HIV services, including ART, adhere to national guidelines.[65 66] The first-line ART regimen is TDF/3TC/dolutegravir; second-line regimens include protease inhibitors. Patients who are virally suppressed are seen in the GHESKIO HIV clinic every 6 months for adherence assessment and medication refills. HIV-1 RNA viral load is measured annually. BP is measured at every clinic visit.

<u>Early HTN treatment vs. SOC procedures</u>: The only difference between study arms is the BP threshold at which amlodipine is initiated. Participants in the early HTN treatment arm will initiate amlodipine 5 mg immediately followed by an increase to 10 mg if SBP >130 mm Hg after 1 month or more. Participants in the SOC arm will initiate the same algorithm but at the standard BP threshold for HTN which is SBP $\geq$140 or DBP $\geq$90 mm Hg in two measurements on different days. There are no known drug-drug interactions between the first-line ART and amlodipine. Haiti's guidelines recommend amlodipine as the first line regimen for diabetic individuals as well.

<u>Clinical management of CVD risk factors and diseases</u>: Any participant diagnosed with a risk factor or disease through study procedures will be escorted to the GHESKIO clinic or affiliated hospital in Port-au-Prince for clinical care and further evaluation by clinicians who are independent of the research team (see Human Subjects). Clinical care is provided free of charge.

Study Outcomes: The primary quantitative outcome is the difference in mean change in SBP (mm Hg) between study arms at 12 months. Secondary outcomes include **viral suppression** defined per WHO guidelines of HIV-1 RNA <1,000 copies/ml and amlodipine and ART **medication adherence** defined as >90% adherence using 3-day pill recalls, as recommended by the ACTG at enrollment, 3, 6, 9 and 12 months.[67] We will assess for serious **adverse events** including DAIDS Grade III-V symptoms of hypotension, peripheral edema and other symptoms related to medications at every study visit (see Human Subjects).[68] **Feasibility and acceptability** will be assessed by enrollment and retention statistics as well as qualitative in-depth interviews among a subset of 30 participants in the early HTN treatment arm and all study providers at 12 months. We will assess participant attitudes towards the study drug amlodipine. Interviews will explore attitudes about initiating amlodipine, positive/negative effects, satisfaction, intent to continue, home BP measurement, and perceived CVD risk. We will interview providers about implementation challenges and unintended consequences. Interviews will be ~45 minutes, conducted in Creole, audio-recorded, transcribed verbatim, and translated into English for analysis using Atlas-ti. Among all participants, we will measure **CVD risk factors** at enrollment (Table 5). In participants >40 years, we will calculate their 10-year AHA atherosclerotic CVD (ASCVD) risk at enrollment.[69] Pre-existing **myocardial and vascular dysfunction** will be measured at enrollment using ECG, echocardiogram, and vascular ultrasound (Sonosite M-Turbo ultra-sound with P21-x probe). Dysfunction is defined as evidence of left ventricular hypertrophy, diastolic and/or systolic dysfunction, ischemia, increased carotid intima media thickness (surrogate for atherosclerosis), and carotid-femoral pulse wave velocity (measure of arterial stiffness) using imaging criteria from the American and European Societies of Echocardiography and Minnesota criteria.[70-73] Dr. Pierre is trained and certified in echocardiography and has experience measuring these parameters with quality assurance from Cornell cardiology in the NHLBI R01 HL143788.

**Table 5: CVD risk factors**
**Factors included in ASCVD:** gender, age, race (white or other), total cholesterol (mg/dL), HDL cholesterol (mg/dL), SBP, received treatment for HTN, diabetes (HbA1c >7 or on medications), smoking
**Other CVD factors**: BMI (kg/m^2), waist circumference, poor diet (WHO STEPS), physical inactivity (WHO STEPS), CD4 count, viral load

4.8 Research staff training and data management

Research staff will be trained in Good Clinical Practice and will receive training for study procedures and a training on AHA/WHO BP measurement guidelines including certification prior to study enrollment and recertification every 6 months. Participants will be identified by an unique study ID number and no participant identifying information will be recorded. Data on research forms will be entered by a data clerk and supervised by Adias Marcelin (Data Manager) into NIH-approved REDCap data management system, which is encrypted, and password protected. Dr. McNairy and Dr. Pierre will oversee data quality control and assurance using CTU procedures. They will review consent documents and REDCap data files for internal validity and completeness. Dr. McNairy will travel monthly to Haiti to meet with the study team and will participate in weekly conference calls with Dr. Pierre.

4.9 Analysis plan and statistical power considerations

Sample size and power: With a sample of 250 participants and 6 time points for BP measurements for each participant, we have 80% power with alpha 0.05 to detect a difference in change in SBP of 4 mm Hg or more between the study arms at 12 months (assuming consistent difference across time and a conservative correlation among measurement of 0.95).[74] We will present summary statistics (e.g., mean and SD of pre, post, and difference within and between arms) in publication for readers and future meta-analyses.

Aim 1: Feasibility will be measured as the proportion of eligible participants willing to participate in the trial and their retention, and the proportion initiating amlodipine. We will compare the mean difference in change in SBP among participants from enrollment to 12 months in each study arm using linear mixed-effects model (LMM) accounting for repeated measures and correlations within subjects, where the time*treatment interaction will serve as "primary" parameter addressing differential slopes for the two arms. We anticipate the two arms will have similar baseline characteristics due to randomization, and therefore the primary analysis will not adjust for baseline variables. If baseline variables are imbalanced, we will adjust factors in regression and report as sensitivity analysis. We will assess incidence of HTN using Kaplan Meier methods. We will analyze dichotomized outcomes (SBP <120 and DBP <80) at all time points via generalized mixed-effects model and at 12 months via Fisher exact tests and logistic regression. In other sensitivity analyses, we will explore the following issues: 1) compliance/adherence (e.g., per protocol analysis); 2) missing data/dropout (via last-value-carried-forward, multiple imputation or inverse-probability weighting); 3) competing risks (e.g., death); 4) joint modeling (of longitudinal and survival data); and 5) changepoint (of treatment initiation in SOC arm).

Aim 2: The proportion of participants in each study arm with viral suppression (HIV-1 RNA < 1000 copies/mL) at 12 months will be compared between study arms using the Fisher exact test; we assume participants who are lost to follow-up or dead at 12 months will have HIV-1 RNA > 1000 copies/mL. Other categorical data including medication adherence, CVD risk factors (e.g. diabetes) will be analyzed similarly. Longitudinal data such as BMI and adherence (continuous or binary/categorical) will be analyzed accounting for repeated measures within participants, via LMM and GLMM as described above. We will also consider Generalized estimating equation-based method (so called, marginal or population-averaged model) and will report consistency or meaningful discrepancy. Acceptability will be assessed in qualitative interviews by Dr. Pierre who is trained in qualitative research using interview information transcribed verbatim, translated into English, and then coded for analysis using a thematic coding scheme. We will also describe adverse events among all participants.

4.10 Timeline

This study will be conducted over two years (8 quarters). In Q1, we will finalize study operating procedures, data collection tools, and staff training. Screening, recruitment, and enrollment will be conducted in Q2-3. Participants will be followed for 12 months from Q2 to Q7. In-depth interviews will be conducted at 12-month clinic visits during Q6-7. In Q7-8, we will finalize data analysis and dissemination.

4.11 Potential challenges and solutions

Insufficient number of eligible individuals to meet target: We will monitor enrollment weekly and expand recruitment to other GHESKIO-affiliated clinics if needed. Missing data and migration: GHESKIO's CTU has a history of >98% retention in clinical trials. We will conduct the 12-month questionnaire by phone among those who cannot return. Since the treatment initiation threshold varies across study arms, blinding is not feasible. We will explore the use of placebo and blinding for the future trial.

4.12 Summary and future directions

There is an urgent need for CVD prevention among PLWH with elevated BP who have alarmingly high risk of CVD events and related mortality. This study is the first step towards evaluating the safety and benefits of earlier HTN treatment among PLWH and will inform a larger trial powered for CVD events. If we demonstrate feasibility and find benefit, the larger trial to follow may shift the paradigm of antihypertensive treatment for CVD prevention in PLWH, both in the US and abroad.

4.13 Data Safety and Monitoring Board and Procedures:

The following monitoring steps will be taken to monitor and ensure the safety of research volunteers:

- The study and consent forms must be approved by Institutional Review Boards in Haiti and Weill Cornell prior to the start of the trial.
- The Principle Investigator will receive a weekly log of adverse events and will review this log each week with the local study team and international collaborators.
- The study team is responsible for reporting all severe Grade III-V adverse events or death suffered by study participants to the IRBs and DSMB within 24 hours of knowing of the event.
- The Principle Investigator must renew IRB approval every year through submission of an annual update including a description of all adverse events.

A Data Safety Monitoring Board (DSMB) will ensure the safety of trial participants through monitoring study procedures, implementation, study findings and review of all adverse events including social harms

The DSMB will meet before enrollment starts to review the study protocol for protection of human participants. The DSMB will then meet every six months. Prior to their meeting, they will receive summary reports of study progress, all adverse events including loss of confidentiality, stigma, social harms and Grade III-V adverse events reported by a study participant. Any severe adverse event, Grade III or IV or death will be reported to the DSMB Chair, within 24 hours of documentation by study staff, who will also report it to Dr. McNairy (PI) and Dr. Pape (Haitian Site PI). After each meeting, the DSMB will provide a written report on the status of the study and their recommendations for any modifications of the study. This report will be provided to the PI and IRBs. The DSMB had the right to halt the study for safety concerns at any time.

5 REFERENCES

1. Nations U. United Nations Human Development Index. http://hdr.undp.org/en/content/table-1-human-development-index-and-its-components 2014 [accessed December 20, 2014.
2. UNAIDS. Haiti: Epidemiological Fact Sheet. http://www.unaids.org/en/dataanalysis/datatools/aidsinfo 2014 [accessed Accessed December 12, 2014.
3. UNICEF. Adolescents map HIV risk, part of a holistic approach to treating HIV/AIDS in Haiti. http://www.unicef.org/infobycountry/haiti_65428.html [
4. Institut Haïtien de l'Enfance (IHE) and ICF. Enquête Mortalité, Morbidité et Utilisation des Services (EMMUS-VI 2016-2017). Pétion-Ville, Haïti, and Rockville, Maryland, USA, 2018.
5. Evaluation IfHMa. Health Data Haiti. http://www.healthdata.org/haiti 2017 [accessed September 4 2017.
6. Roth GA, Johnson C, Abajobir A, et al. Global, Regional, and National Burden of Cardiovascular Diseases for 10 Causes, 1990 to 2015. *Journal of the American College of Cardiology* 2017;70(1):1-25. doi: 10.1016/j.jacc.2017.04.052
7. WHO. WHO Noncommunicable diseases country profile in Haiti, 2014. www.who.int/nmh/countries/hti_en.pd: WHO; 2017 [accessed September 12 2017.
8. (IHME) IfHMaE. GBD Compare Data Visulation. Available from: http://vizhub.healthdata.org/gbd-compare. Seattle, WA: IHME, University of Washington; 2017 [accessed September 20 2017.
9. Tymejczyk O, McNairy ML, Petion JS, et al. Hypertension prevalence and risk factors among residents of four slum communities: population-representative findings from Port-au-Prince, Haiti. *J Hypertens* 2019;37(4):685-95. doi: 10.1097/HJH.0000000000001966 [published Online First: 2019/03/01]
10. McNairy ML, Tymejczyk O, Rivera V, et al. High Burden of Non-communicable Diseases among a Young Slum Population in Haiti. *Journal of urban health : bulletin of the New York Academy of Medicine* 2019 doi: 10.1007/s11524-019-00368-y [published Online First: 2019/06/21]
11. Lavados PM, Hennis AJ, Fernandes JG, et al. Stroke epidemiology, prevention, and management strategies at a regional level: Latin America and the Caribbean. *The Lancet Neurology* 2007;6(4):362-72. doi: 10.1016/s1474-4422(07)70003-0 [published Online First: 2007/03/17]
12. UNAIDS. UNAIDS HIV and AIDS Estimates (2016): Haiti. Available from: http://www.unaids.org/en/regionscountries/countries/haiti 2016 [accessed October 3 2016.
13. Severe P, Juste MA, Ambroise A, et al. Early versus standard antiretroviral therapy for HIV-infected adults in Haiti. *N Engl J Med* 2010;363(3):257-65. doi: 10.1056/NEJMoa0910370
14. Koenig SP, Dorvil N, Devieux JG, et al. Same-day HIV testing with initiation of antiretroviral therapy versus standard care for persons living with HIV: A randomized unblinded trial. *PLoS Med* 2017;14(7):e1002357. doi: 10.1371/journal.pmed.1002357
15. Reif L, Bertrand R, Luis B. Pape JW, Fitzgerald DW, Abrams EJ, McNairy ML. Community-based adolescent and youth HIV testing and enrollement in HIV care in Haiti. 21st International AIDS Conference. Durban, South Africa, 2016.
16. Pape JW, Jean SS, Ho JL, et al. Effect of isoniazid prophylaxis on incidence of active tuberculosis and progression of HIV infection. *Lancet* 1993;342(8866):268-72.
17. Rouzier V, Severe K, Juste MA, et al. Cholera vaccination in urban Haiti. *Am J Trop Med Hyg* 2013;89(4):671-81. doi: 10.4269/ajtmh.13-0171
18. Rivera V J-JM, Gluck S, Reeder H, Sainristil J, Julma P, Peck M, Joseph P, Ocheretina O, Perodin C, Secours R, Duran-Menicuti M, Hashiguchi L, Cremieux P, Koenig S, Pape J. Diagnostic Yield of active case finding for tuberculosis and HIV at the household level in slums in Haiti. *Int J Tuberc Lung Dis* 2017;21(11):e publication. [published Online First: July 6 2017]
19. Sévère K, Rouzier V, Anglade SB, et al. Effectiveness of oral cholera vaccine in Haiti: 37-month follow-up. *The American journal of tropical medicine and hygiene* 2016;94(5):1136-42.

20. Heidkamp RA, Stoltzfus RJ, Fitzgerald DW, et al. Growth in late infancy among HIV-exposed children in urban Haiti is associated with participation in a clinic-based infant feeding support intervention. *The Journal of nutrition* 2012;142(4):774-80. doi: 10.3945/jn.111.155275
21. Pape JW, Severe PD, Fitzgerald DW, et al. The Haiti research-based model of international public health collaboration: the GHESKIO Centers. *J Acquir Immune Defic Syndr* 2014;65 Suppl 1:S5-9. doi: 10.1097/QAI.0000000000000031
22. WHO. HEARTS Technical Package for Cardiovascular Disease Mangement in Primary Health Care. https://apps.who.int/iris/bitstream/handle/10665/260421/WHO-NMH-NVI-18.2-eng.pdf;jsessionid=78326314BF7E5CF661061B0DA3DAA690?sequence=1. Geneva: WHO, 2018.
23. Health CCfG. Cardiovascular Disease in Haiti: Establishign Hypertension Primary Care Guidelines and Initiating a Longitudinal Cohort Study. Available: https://globalhealth.weill.cornell.edu/sites/default/files/cvd_haiti_meeting_summary_with_htn_primary_care_guidelnes_version_1.0.pdf. Port au Prince, Haiti: Cornell-GHESKIO, 2019:1-12.
24. Ojji DB, Mayosi B, Francis V, et al. Comparison of Dual Therapies for Lowering Blood Pressure in Black Africans. *N Engl J Med* 2019;380(25):2429-39. doi: 10.1056/NEJMoa1901113 [published Online First: 2019/03/19]
25. Flack JM, Sica DA, Bakris G, et al. Management of high blood pressure in Blacks: an update of the International Society on Hypertension in Blacks consensus statement. *Hypertension* 2010;56(5):780-800. doi: 10.1161/HYPERTENSIONAHA.110.152892 [published Online First: 2010/10/06]
26. Jamerson K, Weber MA, Bakris GL, et al. Benazepril plus amlodipine or hydrochlorothiazide for hypertension in high-risk patients. *N Engl J Med* 2008;359(23):2417-28. doi: 10.1056/NEJMoa0806182 [published Online First: 2008/12/05]
27. Medicine WC. Weill Cornell Medicine and GHESKIO Receive Grant to Improve Blood Pressure Control in Haiti: https://news.weill.cornell.edu/news/2019/11/weill-cornell-medicine-and-gheskio-receive-grant-to-improve-blood-pressure-control-in 2019 [accessed November 15 2019.
28. Batavia AS, Severe P, Lee MH, et al. Blood pressure and mortality in a prospective cohort of HIV-infected adults in Port-au-Prince, Haiti. *J Hypertens* 2018;36(7):1533-39. doi: 10.1097/HJH.0000000000001723 [published Online First: 2018/04/11]
29. Armah KA, Chang CC, Baker JV, et al. Prehypertension, hypertension, and the risk of acute myocardial infarction in HIV-infected and -uninfected veterans. *Clin Infect Dis* 2014;58(1):121-9. doi: 10.1093/cid/cit652 [published Online First: 2013/09/26]
30. Goff DC, Jr., Lloyd-Jones DM, Bennett G, et al. 2013 ACC/AHA guideline on the assessment of cardiovascular risk: a report of the American College of Cardiology/American Heart Association Task Force on Practice Guidelines. *Circulation* 2014;129(25 Suppl 2):S49-73. doi: 10.1161/01.cir.0000437741.48606.98 [published Online First: 2013/11/14]
31. Fahme SA, Bloomfield GS, Peck R. Hypertension in HIV-Infected Adults: Novel Pathophysiologic Mechanisms. *Hypertension* 2018;72(1):44-55. doi: 10.1161/HYPERTENSIONAHA.118.10893 [published Online First: 2018/05/20]
32. Nuesch R, Wang Q, Elzi L, et al. Risk of cardiovascular events and blood pressure control in hypertensive HIV-infected patients: Swiss HIV Cohort Study (SHCS). *J Acquir Immune Defic Syndr* 2013;62(4):396-404. doi: 10.1097/QAI.0b013e3182847cd0 [published Online First: 2013/01/05]
33. Xu Y, Chen X, Wang K. Global prevalence of hypertension among people living with HIV: a systematic review and meta-analysis. *J Am Soc Hypertens* 2017;11(8):530-40. doi: 10.1016/j.jash.2017.06.004 [published Online First: 2017/07/12]
34. Ryscavage P, Still W, Nyemba V, et al. Prevalence of Systemic Hypertension Among HIV-Infected and HIV-Uninfected Young Adults in Baltimore, Maryland. *South Med J* 2019;112(7):387-91. doi: 10.14423/SMJ.0000000000001001 [published Online First: 2019/07/10]

35. Shah ASV, Stelzle D, Lee KK, et al. Global Burden of Atherosclerotic Cardiovascular Disease in People Living With HIV. *Circulation* 2018;138(11):1100-12. doi: 10.1161/CIRCULATIONAHA.117.033369 [published Online First: 2018/07/04]
36. Manner IW, Troseid M, Oektedalen O, et al. Low nadir CD4 cell count predicts sustained hypertension in HIV-infected individuals. *J Clin Hypertens (Greenwich)* 2013;15(2):101-6. doi: 10.1111/jch.12029 [published Online First: 2013/01/24]
37. Bautista LE, Vera LM, Arenas IA, et al. Independent association between inflammatory markers (C-reactive protein, interleukin-6, and TNF-alpha) and essential hypertension. *Journal of human hypertension* 2005;19(2):149-54. doi: 10.1038/sj.jhh.1001785 [published Online First: 2004/09/14]
38. Subramanian S, Tawakol A, Burdo TH, et al. Arterial inflammation in patients with HIV. *JAMA* 2012;308(4):379-86. doi: 10.1001/jama.2012.6698 [published Online First: 2012/07/24]
39. Grinspoon S, Carr A. Cardiovascular risk and body-fat abnormalities in HIV-infected adults. *N Engl J Med* 2005;352(1):48-62. doi: 10.1056/NEJMra041811 [published Online First: 2005/01/07]
40. Grinspoon SK. Cardiovascular disease in HIV: traditional and nontraditional risk factors. *Top Antivir Med* 2014;22(4):676-9. [published Online First: 2014/11/15]
41. Williams B, Mancia G, Spiering W, et al. 2018 ESC/ESH Guidelines for the management of arterial hypertension. *European heart journal* 2018;39(33):3021-104.
42. Rapsomaniki E, Timmis A, George J, et al. Blood pressure and incidence of twelve cardiovascular diseases: lifetime risks, healthy life-years lost, and age-specific associations in 1.25 million people. *Lancet* 2014;383(9932):1899-911. doi: 10.1016/S0140-6736(14)60685-1 [published Online First: 2014/06/03]
43. Shen L, Ma H, Xiang MX, et al. Meta-analysis of cohort studies of baseline prehypertension and risk of coronary heart disease. *The American journal of cardiology* 2013;112(2):266-71. doi: 10.1016/j.amjcard.2013.03.023 [published Online First: 2013/04/24]
44. Huang Y, Cai X, Zhang J, et al. Prehypertension and Incidence of ESRD: a systematic review and meta-analysis. *American journal of kidney diseases : the official journal of the National Kidney Foundation* 2014;63(1):76-83. doi: 10.1053/j.ajkd.2013.07.024 [published Online First: 2013/10/01]
45. Huang Y, Wang S, Cai X, et al. Prehypertension and incidence of cardiovascular disease: a meta-analysis. *BMC medicine* 2013;11:177. doi: 10.1186/1741-7015-11-177 [published Online First: 2013/08/07]
46. Vasan RS, Larson MG, Leip EP, et al. Impact of high-normal blood pressure on the risk of cardiovascular disease. *N Engl J Med* 2001;345(18):1291-7. doi: 10.1056/NEJMoa003417 [published Online First: 2002/01/17]
47. Julius S, Nesbitt SD, Egan BM, et al. Feasibility of treating prehypertension with an angiotensin-receptor blocker. *N Engl J Med* 2006;354(16):1685-97. doi: 10.1056/NEJMoa060838 [published Online First: 2006/03/16]
48. Dzau V. The cardiovascular continuum and renin-angiotensin-aldosterone system blockade. *J Hypertens Suppl* 2005;23(1):S9-17. [published Online First: 2005/04/12]
49. Folkow B. Physiological aspects of primary hypertension. *Physiol Rev* 1982;62(2):347-504. doi: 10.1152/physrev.1982.62.2.347 [published Online First: 1982/04/01]
50. Nissen SE, Tuzcu EM, Libby P, et al. Effect of antihypertensive agents on cardiovascular events in patients with coronary disease and normal blood pressure: the CAMELOT study: a randomized controlled trial. *JAMA* 2004;292(18):2217-25. doi: 10.1001/jama.292.18.2217 [published Online First: 2004/11/13]
51. Heart Outcomes Prevention Evaluation Study I, Yusuf S, Sleight P, et al. Effects of an angiotensin-converting-enzyme inhibitor, ramipril, on cardiovascular events in high-risk patients. *N Engl J Med* 2000;342(3):145-53. doi: 10.1056/NEJM200001203420301 [published Online First: 2000/01/20]
52. Xie X, Atkins E, Lv J, et al. Effects of intensive blood pressure lowering on cardiovascular and renal outcomes: updated systematic review and meta-analysis. *Lancet* 2016;387(10017):435-43. doi: 10.1016/S0140-6736(15)00805-3 [published Online First: 2015/11/13]

53. Lv J, Ehteshami P, Sarnak MJ, et al. Effects of intensive blood pressure lowering on the progression of chronic kidney disease: a systematic review and meta-analysis. *CMAJ* 2013;185(11):949-57. doi: 10.1503/cmaj.121468 [published Online First: 2013/06/27]
54. Group SR, Wright JT, Jr., Williamson JD, et al. A Randomized Trial of Intensive versus Standard Blood-Pressure Control. *N Engl J Med* 2015;373(22):2103-16. doi: 10.1056/NEJMoa1511939 [published Online First: 2015/11/10]
55. Whelton PK, Carey RM, Aronow WS, et al. 2017 ACC/AHA/AAPA/ABC/ACPM/AGS/APhA/ASH/ASPC/NMA/PCNA Guideline for the Prevention, Detection, Evaluation, and Management of High Blood Pressure in Adults: Executive Summary: A Report of the American College of Cardiology/American Heart Association Task Force on Clinical Practice Guidelines. *Circulation* 2018;138(17):e426-e83. doi: 10.1161/CIR.0000000000000597 [published Online First: 2018/10/26]
56. Fox CS, Coady S, Sorlie PD, et al. Trends in cardiovascular complications of diabetes. *JAMA* 2004;292(20):2495-9. doi: 10.1001/jama.292.20.2495 [published Online First: 2004/11/25]
57. Simonyi G. Benefits of Fixed Dose Combination of Ramipril/Amlodipine in Hypertensive Diabetic Patients: A Subgroup Analysis of RAMONA Trial. Chin Med J (Engl)2016:1224-8.
58. Nissen SE, Tuzcu EM, Peter L, et al. Effect of Antihypertensive Agents on Cardiovascular Events in Patients With Coronary Disease and Normal Blood Pressure: The CAMELOT Study: A Randomized Controlled Trial. *JAMA* 2019;292(18):2217-25. doi: 10.1001/jama.292.18.2217
59. WHO. WHO STEPS Surveillance Manual. Accessed from: http://www.who.int/chp/steps/manual/en/index3.html Geneva: WHO; 2017 [accessed May 30 2017.
60. Dyer AR, Cutter GR, Liu KQ, et al. Alcohol intake and blood pressure in young adults: the CARDIA Study. *Journal of clinical epidemiology* 1990;43(1):1-13.
61. Pletcher MJ, Varosy P, Kiefe CI, et al. Alcohol consumption, binge drinking, and early coronary calcification: findings from the Coronary Artery Risk Development in Young Adults (CARDIA) Study. *Am J Epidemiol* 2005;161(5):423-33. doi: 10.1093/aje/kwi062
62. Halanych JH, Safford MM, Kertesz SG, et al. Alcohol consumption in young adults and incident hypertension: 20-year follow-up from the Coronary Artery Risk Development in Young Adults Study. *Am J Epidemiol* 2010;171(5):532-9. doi: 10.1093/aje/kwp417
63. Abuse NIoD. NIDA Resource Guide: Screening for Drug Use in General Medicine Settings, the NIDA Quick Screen. Available from: https://www.drugabuse.gov/publications/resource-guide-screening-drug-use-in-general-medical-settings/nida-quick-screen Bethesda, MD: NIDA; 2017 [accessed October 30 2017.
64. Bull FC, Maslin TS, Armstrong T. Global physical activity questionnaire (GPAQ): nine country reliability and validity study. *Journal of physical activity & health* 2009;6(6):790-804.
65. Ministere de la Sante Publique et de la Population H. Manuel de Norms de Prise en Charge, Clinique et Therapeutique des Adultes et Adolescents Vivant Avec Le VIH/SIDA. file:///C:/Users/mam9365/Downloads/NORMES%20PEC%2029%20octobre%202013%20(1).pdf. Port au Prince: MSPP, 2013.
66. WHO. Update of Recommendations on First- and Second-line Antiretroviral Regimens, July 2019. https://apps.who.int/iris/bitstream/handle/10665/325892/WHO-CDS-HIV-19.15-eng.pdf?ua=1. Geneva: WHO, 2019.
67. Chesney MA, Ickovics JR, Chambers DB, et al. Self-reported adherence to antiretroviral medications among participants in HIV clinical trials: the AACTG adherence instruments. Patient Care Committee & Adherence Working Group of the Outcomes Committee of the Adult AIDS Clinical Trials Group (AACTG). *AIDS Care* 2000;12(3):255-66. doi: 10.1080/09540120050042891 [published Online First: 2000/08/06]
68. U.S. Department of Health and Human Services NIoH, National Institute of Allergy and Infectious Diseases. Division of AIDS. Division of AIDS (DAIDS) Table for Grading the Severity of Adult and Pediatric Adverse

Events, Corrected Version 2.1. [July 2017]. Available from: https://rsc.niaid.nih.gov/sites/default/files/daidsgradingcorrectedv21.pdf 2017 [accessed 2019 November 1.
69. Association ACoCAH. ASCVD Risk Estimator Web Tool available at http://tools.acc.org/ASCVD-Risk-Estimator-Plus/#!/calculate/estimate/ 2019 [accessed November 22 2019.
70. Lang RM, Badano LP, Mor-Avi V, et al. Recommendations for cardiac chamber quantification by echocardiography in adults: an update from the American Society of Echocardiography and the European Association of Cardiovascular Imaging. *European heart journal cardiovascular Imaging* 2015;16(3):233-70. doi: 10.1093/ehjci/jev014
71. Prineas R, Crow R, Blackburn H. The Minnesota code manual of electrocardiographic findings. John Wright-PSG. *Inc Littleton, MA* 1982
72. Stein JH, Korcarz CE, Hurst RT, et al. Use of carotid ultrasound to identify subclinical vascular disease and evaluate cardiovascular disease risk: a consensus statement from the American Society of Echocardiography Carotid Intima-Media Thickness Task Force. Endorsed by the Society for Vascular Medicine. *J Am Soc Echocardiogr* 2008;21(2):93-111; quiz 89-90. doi: 10.1016/j.echo.2007.11.011 [published Online First: 2008/02/12]
73. Van Bortel LM, Laurent S, Boutouyrie P, et al. Expert consensus document on the measurement of aortic stiffness in daily practice using carotid-femoral pulse wave velocity. *Journal of hypertension* 2012;30(3):445-8. doi: 10.1097/HJH.0b013e32834fa8b0 [published Online First: 2012/01/27]
74. Diggle P, Diggle PJ, Heagerty P, et al. Analysis of longitudinal data: Oxford University Press 2002.
