# Supplementary material for: Treatment of early hypertension among persons living with HIV in Haiti: Protocol for a randomized controlled trial
Source: PLoS One. 2021 Aug 5;16(8):e0254740. doi: 10.1371/journal.pone.0254740 (PMC8341523; doi:10.1371/journal.pone.0254740)
Supplement: S2 File — (PDF) [file pone.0254740.s003.pdf]

**SEKSYON A: ENFÒMASYON SOU PATISIPAN AN**<br>RANPLI TOUT ENFÒMASYON ANBA YO ANVAN OU KÒMANSE KEKSYONÈ A				
	Variab	Keksyon	Repons	QC
A1	entry_PID	Study PID:	□□□□□□□□	
Make lè keksyonè a kòmanse. Lè ou fini, make lè ou fini a tou.				
A2	entry_staff	Inisyal Anplwaye a	□□	
A3	entry_date	Dat keksyonè a pase	□□ □□ □□□□<br>JOU MWA LANE	
A4	entry_starttime	Lè keksyonè a kòmanse (24è)	□□:□□	

Remak pou anketè a:

1. Tout sa yo ki **EKRI ANGWO LÈT NWA FONSE** ta dwe li mo pou mo epi byen fò pou patisipan an.
2. Enstriksyon pou *anketè* yo ekri an *italic* epi yo pa dwe li fò.

ENTWODIKSYON KEKSYONÈ A

Bonjou non mwen se _______. Map travay nan Sant GHESKIO kòm _________. Mèsi paske ou dakò patisipe nan ankèt sa. Nou espere enfòmasyon nou kolekte yo ap ede nou konprann tansyon wo kay moun ki enfekte ak VIH pou nou jwenn fason pou nou anpeche ak trete tansyon wo ak maladi kè an Ayiti.

Mwen pral poze ou kèk keksyon pandan apeprè 30 minit sou divès bagay tankou sou sa w fè pou travay, sou zafè lasante ou, tankou si w fimen epi bay kòw aktivite fizik, sou kòman ou santiw, sou kalite lavi ou, ak kominote ou.

Kèk nan keksyon mwen pral poze w yo kapab fè w santi ou pa alèz pou reponn. Ou ka chwazi pa reponn nenpòt keksyon ou pa santi w alèz pou reponn.

Tanpri sonje tout enfòmasyon ou ban nou ap rete sekrè epi yo pap pataje ak nenpòt moun andeyò ekip etid la. Mete w alèz pou w ka reponn keksyon yo avèk onètete jan ou kapab. Si nenpòt nan keksyon sa yo pa klè, tanpri kanpe mwen, epi mwen pral ba w plis eksplikasyon. Sonje byen, pa gen okenn repons ki bon oswa ki pa bon.

Èske w gen yon keksyon anvan nou kòmanse?

Reponn nenpòt enkyetid oswa kesyon anvan ou kontinye.

**SEKSYON B : ENFÒMASYON SOSYODEMOGRAFIK**<br><br>**Mwen pral kòmanse poze w kèk keksyon sou ou menm.**				
	**Variab**	**Keksyon**	**Repons**	QC
B1	sex	Ki sèks ou?	☐1.Gason<br>☐ 2. Fi<br>☐ 3. Lòt<br>☐ 99. Pa konnen, refize reponn	
B2	age	Li laj ou (lane)?	__________ lane	
B3	ses_education	Ki pi gwo klas ou fè/rive lekòl?<br><br>*Li chwa yo byen fò pou patisipan an*	☐ 1. Okenn<br>☐ 2. Primè<br>☐ 3. Segondè<br>☐ 4. Pi wò pase segondè<br>☐ 99. Unknown / Refuse to answer, enkoni, refize reponn	

B4	ses_marital	Eske ou marye (Ki estati sivil ou pou kounye a)?	☐ 1. Single, Selibatè<br>☐ 2. Living together, Plase/Viv avèk<br>☐ 3. Married, Marye<br>☐ 4. Widowed, Vèf/Vèv<br>☐ 5. Divorced/separated, Divòse/Separe<br>☐ 99. Pa konnen, refize reponn	
B5	ses_occupation	Ki travay ou fè jodia?<br><br>*Si yon moun ap etidye epi lap travay, tyeke anplwaye.*	☐ 1. Komès<br>☐ 2. Anplwaye<br>☐ 3. Etidyan<br>☐ 4. Jere kay la<br>☐ 5. Retrete<br>☐ 6. Pap travay<br>☐ 99. Pa konnen, refize reponn	
B6	ses_income	Konbyen lajan ou touche pa jou?<br><br>*Li chwa yo byen fò pou patisipan an. Si patisipan an bay lajan li touche a an goud, konvèti li an Dola Ayisyen: 1 HD = 5 goud.*	☐ 1. Okenn<br>☐ 2. Pipiti pase 10 HD/jou<br>☐ 3. 11-20 HD/jou<br>☐ 4. 21-200 HD/jou<br>☐ 5. Plis pase 200 HD/jou<br>☐ 99. Pa konnen, refize reponn	
**ENDEKS POVETE MULTIDIMENSYONÈL**<br>**Koulye a, mwen pral poze w kesyon sou kay ou ak lakay ou, pou wè si ou gen ase resous pou sipòte sante ou. Kesyon sa yo aplike a nenpòt moun k ap viv nan kay moun nan (fanmi, zanmi, kolokasyon, elatriye)**				
B7	ses_living_children	Konbyen pitit ki vivan ou genyen (kelkeswa laj li/yo)?	___________ timoun vivan	
B8	ses_undernourish	Nan kay ou, èske gen yon moun (granmoun oswa timoun) ki twò piti oswa ki pa gen ase manje?	☐ 1. Wi<br>☐ 2. Non<br>☐ 99. Pa konnen, refize reponn	
B9	ses_childdeath	Nan kay ou, èske gen timoun <18 ane ki mouri nan senk dènye ane yo?<br><br>*Si pa gen timoun, make non*	☐ 1. Wi<br>☐ 2. Non<br>☐ 99. Pa konnen, refize reponn	
B10	ses_primaryschool	Nan kay ou, èske gen moun ki te konplete omwen lekòl primè?	☐ 1. Wi<br>☐ 2. Non<br>☐ 99. Pa konnen, refize reponn	
B11	ses_childschool	Nan kay ou, èske gen nenpòt timoun ki gen laj 5-13 ane ki pa ale lekòl?	☐ 1. Wi<br>☐ 2. Non<br>☐ 99. Pa konnen, refize reponn	
B12	ses_cook	Nan kay ou, ki kalite gaz ki itilize pou kwit manje?	☐ 1. Fimye, bwa, chabon, chabon, oswa zèb<br>☐ 2. Elektrisite, gaz, kewozen<br>☐ 88. Lòt<br>☐ 99. Pa konnen, refize reponn	
B13	ses_sanitation	Nan kay ou, ki kalite twalèt ki itilize?	☐ 1. Latrin ki pataje ak plizye moun, bokit, twalèt pandye, twalèt a tè a nan jaden<br>☐ 2. Flush toilet, piped sewer system, flush pit latrine, ventilated improved pit latrine<br>Twalèt WC ki flush, sistèm egou kanalize, latrin twou san fon, latrin amelyore<br>☐ 88. Lòt<br>☐ 99. Pa konnen, refize reponn	
B14	ses_water	Nan kay ou, ki sous prensipal dlo potab?	☐ 1. Pi ki pa kouvri, dlo ki soti nan yon ti sous dlo, dlo sifas (la rivyè, sous, lak)<br>☐ 2. Dlo tiyo, ponp piblik, twou dlo, pi ki kouvri, dlo lapli, dlo trete nan boutèy<br>☐ 88. Other, lòt<br>☐ 99. Pa konnen, refize reponn	

B15	ses_electricity	Èske kay ou gen elektrisite?	☐ 1. Wi<br>☐ 2. Non<br>☐ 99. Pa konnen, refize reponn	
B16	ses_housing	Nan kay ou a, ak kisa atè a fèt?	☐ 1. Tè, sab, fimye ak bwa, planch bwa<br>☐ 2. Bwa poli, vinil, mozayik seramik, siman, tapi/kapèt<br>☐ 99. Pa konnen, refize reponn	
B17	ses_items	Èske kay ou gen nenpòt nan bagay sa yo mwen pral di w la?<br><br>*Make tout sa ki wi*	☐ 1. Radyo<br>☐ 2. Frijidè<br>☐ 3. Televizyon<br>☐ 4. Fou / recho<br>☐ 5. Telefòn selilè<br>☐ 6. Bisiklèt<br>☐ 7. Motosiklèt<br>☐ 8. Machin oswa kamyon<br>☐ 99. Pa konnen, refize reponn	
**SEKSYON C: KOPÒTMAN SANTE KI GEN RAPÒ AK MALADI KÈ**				
**A**LIMANTASYON: **Kounye a mwen pral poze w keksyon konsènan fwi ak legim ou abitye manje. Mwen pral poze w keksyon tou sou sou kantite sèl ou sèvi nan manje.**				
C1	diet_eatout_0M	Nan yon semèn abityèl, konbyen jou ou manje manje kwit nan men machann, yon restoran oubyen yon kafeterya?	☐ 1. 0-1 jou<br>☐ 2. 2-3 jou<br>☐ 3. 4-5 jou<br>☐ 4. Plis pase 5 jou<br>☐ 99. Pa konnen, refize reponn	
C2	diet_friedfood_0M	Nan yon semèn abityèl, konbyen jou ou manje yon manje ki gen ladan l manje ki fri nan lwil, tankou bannann peze, akra, oubyen griyo?	☐ 1. 0-1 jou<br>☐ 2. 2-3 jou<br>☐ 3. 4-5 jou<br>☐ 4. Plis pase 5 jou<br>☐ 99. Pa konnen, refize reponn	
C3	diet_fruit_serv_0M	Ki kantite fwi ou manje nan yon jou nòmal (ki nan abitid ou)? Lè nou pale de fwi, sa vle di ½ kèp fwi yo oubyen yon fwi ki pa two gwo tankou zaboka oubyen mango. Ji pa ladan l	*Antre yon kantite: # kèp*<br><br>___________	
C4	diet_veg_serv_0M	Ki kantite legim ou manje nan yon jou nòmal / abityèl? Lè mwen pale de legim mwen vle di ½ kèp pòmdetè, joumou, oubyen kawòt oubyen 1 kèp fèy legim vèt tankou epina. Mwen pa pale de bannann peze, sòs pwa oubyen diri kole.	*Antre yon kantite: # kèp*<br><br>___________	
C5	diet salt_use_0M	Konbyen fwa moun ki fè manje oubyen moun ki prepare manje lakay ou itilize sèl kwizin oubyen asezònman ki gen sèl tankou Magi, sèl lay, sèl zonyon, sòy sòs oubyen sòs pwason?	☐ 1.Toujou/Souvan<br>☐ 2. Kèk fwa<br>☐ 3. Raman/Jamè<br>☐ 99. Pa konnen, refize reponn	
C6	diet salt_use2_0M	Konbyen fwa ou ajoute sèl nan manje w oubyen asezònman ki gen sèl tankou Magi, sèl lay, sèl zonyon, sòy sòs, oubyen sòs pwason anvan ou manje l oubyen pandan wap manje l?	☐ 1.Toujou/Souvan<br>☐ 2. Kèk fwa<br>☐ 3. Raman/Jamè<br>☐ 99. Pa konnen, refize reponn	
AKTIVITE FIZIK: **Kounye a, mwen pral poze ou keksyon sou kantite aktivite fizik ou fè, ki gen ladan travay ou ak deyò nan travay ou.**				
C7	activity_type_0M	Eske wap fè travay manyèl? Lè map di travay manyèl, m vle di travay ki fè souf ou wo, fè kè w bat fò pandana plis pase 10 minit. Tankou konstriksyon oubyen agrikiltè.	☐ 1.Yes, Wi<br>☐ 2.No, Non<br>☐ 99. Pa konnen, refize reponn	
C8	activity_moderate_0M	Andeyò travay ou, eske ou fè lòt kalite espò, aktivite fizik oswa aktivite pou pran plezi w ki fè souf ou wo oubyen kè w bat fò (tankou mache vit oubyen netwaye lakay w) pou omwen 150 minit (2 ½ èdtan) nan yon semèn?	☐ 1.Yes, Wi<br>☐ 2.No, Non<br>☐ 99. Pa konnen, refize reponn	

SÈVI AK TABAK **Kounye a mwen ta renmen pozew keksyon sou itilizasyon tabak w.**				
C9	tobacco_ever_0M	Eske ou te janm fimen pou pipiti 100 sigarèt nan tout vi ou?	☐ 1.Yes, Wi<br>☐ 2.No, Non<br>☐ 3. Pa konnen<br>☐ 99. Pa konnen, refize reponn	
C10	tobacco_current_0M	Eske ou fimen nenpòt pwodwi tabak kounye a?	☐ 1. Wi<br>☐ 2. Non=>*Ale ale nan keksyon C12*<br>☐ 99. Pa konnen, refize reponn	
C11	tobacco_daily_0M	Eske ou konn fimen nenpòt pwodwi tabak chak jou kounye a? Lè mwen di chak jou, mwen vle di preske chak jou pou omwen yon lane.	☐ 1.Yes, Wi<br>☐ 2.No, Non<br>☐ 3. Pa konnen<br>☐ 99. Pa konnen, refize reponn	
BWASON KI GEN ALKÒL: **Kounye a mwen ta renmen poze ou keksyon sou itilizasyon alkòl oswa bweson ou.**				
C12	alcohol_currently_0M	Pandan 12 mwa ki sot pase yo, eske ou te bwè alkòl oubyen pwodwi alkòl tankou kleren epi tranpe?	☐ 1. Wi<br>☐ 2. Non=>*Ale ale nan keksyon D1*<br>☐ 99. Pa konnen, refize reponn	
C13	alcohol_currentlyany_0M	Nan dènye 12 mwa ki sot pase yo, konbyen fwa ou te bwè yon bweson ki gen alkòl? Lè mwen di “bweson ki gen alkòl”, mwen vle di yon boutèy 12-ons oubyen yon vè byè, yon vè 5-ons diven, oswa yon bwasson ki gen 1 ti vè likè kòm rhum oubyen kleren.<br><br>.<br><br>*Li chwa yo fò pou patisipan an. Chwazi sèlman yon sèl chwa.*	☐ 1. Chak jou<br>☐ 2. 5 - 6 fwa pa semèn<br>☐ 3. 3 - 4 fwa pa semèn<br>☐ 4. 2 fwa pa semèn<br>☐ 5. Yon sèl fwa pa semèn<br>☐ 6. 2 - 3 fwa pa mwa<br>☐ 7. Yon sèl fwa pa mwa<br>☐ 8. 3 – 11 fwa nan lane ki sot pase a<br>☐ 9. 1- 2 fwa nan lane ki sot pase a<br>☐ 99. Pa konnen, refize reponn	
C14	alcohol_quantity_0M	Pandan 12 mwa ki sot pase yo, konbyen bweson ki gen alkòl ou te ka bwè nan jou ou tap bwè alkòl?	*Antre yon chif ant 1-30*<br><br>____________<br><br>*Si patisipan se yon gason, ale nan keksyon C4.*<br>*Si patisipan se yon fi, ale nan keksyon C5.*	
C15	alcohol_max_0M	Pandan 12 mwa ki sot pase yo, konbyen fwa w te bwè 5 oswa plis bweson ki gen nenpòt kalite alkòl nan yon peryòd 2 èdtan? Sa vle di 5 kanèt oubyen boutèy 12-ons byè, 5 vè 5-ons diven oswa 5 bweson ki gen yon sèl ti vè likè tankou rhum oswa kleren.	*Antre yon chif ant 1-30*<br><br>_________	
C16	alcohol_max_0M	Pandan 12 mwa ki sot pase yo, konbyen fwa w te bwè 5 oswa plis bweson ki gen nenpòt kalite alkòl nan yon peryòd 2 èdtan? Sa vle di 5 kanèt oubyen boutèy 12-ons byè, 5 vè 5-ons diven oswa 5 bweson ki gen yon sèl ti vè likè tankou rhum oswa kleren.	*Antre yon chif ant 1-30*<br><br>________	

SESKYON D: ADERANS AK MEDIKAMAN ARV YO (Enstriman mezi ACTG pou aderans, FANMI)
Kounye a mwen pral poze w kèk keksyon sou kijan ou pran medikaman ARV ou yo.

Pifò moun ki gen VIH gen anpil grenn pou yo pran nan diferan moman pandan jounen an.
Anpil moun jwenn li difisil pou yo toujou sonje pran grenn yo:
· Gen kèk moun ki okipe epi bliye pote grenn yo avèk yo.
· Gen kèk moun ki deside sote dòz pou fè pou evite efè segondè oswa jis pa pran grenn jou sa a.

Nou bezwen konprann ki jan moun ki gen VIH reyèlman ap degaje yo ak grenn yo. Tanpri, di nou kijan sa ye pou ou kounye a. Pa enkyete w pou di nou ke ou pa pran tout grenn ou yo. Nou bezwen konnen kijan sa ye reyèlman, pa di nou sa ou panse nou "vle tande."

**Kounye a mwen pral poze w kèk keksyon sou kijan ou pran medikaman ARV ou yo.**				
	Variab	Keksyon	Repons	QC
D1	hiv_medmiss_0M	Nan 4 jou pase yo, konbyen jou ou te pat pran tout dòz medikaman ARV ou yo ?	☐ 0. Okenn<br>☐ 1. 1 jou<br>☐ 2. 2 jou<br>☐ 3. 3 jou<br>☐ 4. 4 jou	
D2	hiv_medwkend_0M	Kèk moun kon di yo konn mal pou sonje pran medikaman yo nan wikenn. Eske ou te bliye pran medikaman ARV ou yo samedi oubyen dimanche ki sot pase a ?	☐ 1. Wi<br>☐ 2. Non	
**Pandan mwa ki sot pase a, konbyen fwa ou pa te pran medikaman ARV ou yo pou yon nan rezon sa yo:**				

			Jamè	Raman	Kèk fwa	Souvan
D3	hiv_away_0M	Ou pat lakay ou?	0	1	2	3
D4	hiv_forgot_0M	Ou te bliye?	0	1	2	3
D5	hiv_pills_0M	Te gen twòp grenn pou pran?	0	1	2	3
D6	hiv_see_0M	Ou te pè efè segondè, oswa ou te santi tankou medikaman yo te toksik / danjere?	0	1	2	3
D7	hiv_stigma_0M	Pat vle lòt moun wè ou pran medikaman?	0	1	2	3
D8	hiv_sick_0M	Te santi ou pat byen oswa ou te malad?	0	1	2	3
D9	hiv_depress_0M	Te santi deprime oswa akable?	0	1	2	3
D10	hiv_ranout_0M	Ou pat gen grenn / medikaman ou te fini?	0	1	2	3
D11	hiv_good_0M	Ou te santi w byen?	0	1	2	3
D12	hiv_religion_0M	Kwayans relijyè?	0	1	2	3

**Keksyonè a fini la. Mèsi paske w te patisipe nan ankèt sa. Eske ou gen nenpòt keksyon pou mwen?**
☐☐:☐☐<br><br>entry_endtime

Remak pou anketè a:
1. Tout sa yo ki EKRI ANGWO LÈT NWA FONSE ta dwe li mo pou mo epi byen fò pou patisipan nan ankèt la.
2. Enstriksyon pou anketè yo ekri an italic epi yo pa dwe li fò.

ENTWODIKSYON KEKSYONÈ A

Bonjou non mwen se _______. Map travay nan Sant GHESKIO kòm _________. Mèsi paske ou dakò patisipe nan ankèt sa. Nou espere enfòmasyon nou kolekte yo ap ede nou konprann tansyon wo kay moun ki enfekte ak VIH pou nou jwenn fason pou nou anpeche ak trete tansyon wo ak maladi kè an Ayiti.

Mwen pral poze ou kèk keksyon pandan apeprè 30 minit sou divès bagay tankou sou sa w fè pou travay, sou zafè lasante ou, tankou si w fimen epi bay kòw aktivite fizik, sou kòman ou santiw, sou kalite lavi ou, ak kominote ou.

Kèk nan keksyon mwen pral poze w yo kapab fè w santi ou pa alèz pou reponn. Ou ka chwazi pa reponn nenpòt keksyon ou pa santi w alèz pou reponn.

Tanpri sonje tout enfòmasyon ou ban nou ap rete sekrè epi yo pap pataje ak nenpòt moun andeyò ekip etid la. Mete w alèz pou w ka reponn keksyon yo avèk onètete jan ou kapab. Si nenpòt nan keksyon sa yo pa klè, tanpri kanpe mwen, epi mwen pral ba w plis eksplikasyon. Sonje byen, pa gen okenn repons ki bon oswa ki pa bon.

Èske w gen yon keksyon anvan nou kòmanse?

Reponn nenpòt enkyetid oswa kesyon anvan ou kontinye.

SEKSYON B: ADERANS AK MEDIKAMAN ARV YO (Enstriman mezi ACTG pou aderans, FANMI)
Kounye a mwen pral poze w kèk keksyon sou kijan ou pran medikaman ARV ou yo.

Pifò moun ki gen VIH gen anpil grenn pou yo pran nan diferan moman pandan jounen an.
Anpil moun jwenn li difisil pou yo toujou sonje pran grenn yo:
· Gen kèk moun ki okipe epi bliye pote grenn yo avèk yo.
· Gen kèk moun ki deside sote dòz pou fè pou evite efè segondè oswa jis pa pran grenn jou sa a.

Nou bezwen konprann ki jan moun ki gen VIH reyèlman ap degaje yo ak grenn yo. Tanpri, di nou kijan sa ye pou ou kounye a. Pa enkyete w pou di nou ke ou pa pran tout grenn ou yo. Nou bezwen konnen kijan sa ye reyèlman, pa di nou sa ou panse nou "vle tande."

**Kounye a mwen pral poze w kèk keksyon sou kijan ou pran medikaman ARV ou yo.**				
	Variab	Keksyon	Repons	QC
B1	hiv_medmiss	Nan 4 jou pase yo, konbyen jou ou te pat pran tout dòz medikaman ARV ou yo ?	☐ 0. Okenn<br>☐ 1. 1 jou<br>☐ 2. 2 jou<br>☐ 3. 3 jou<br>☐ 4. 4 jou	
B2	hiv_medwkend	Kèk moun kon di yo konn mal pou sonje pran medikaman yo nan wikenn. Eske ou te bliye pran medikaman ARV ou yo samedi oubyen dimanche ki sot pase a ?	☐ 1. Wi<br>☐ 2. Non	

Pandan mwa ki sot pase a, konbyen fwa ou pa te pran medikaman ARV ou yo pou yon nan rezon sa yo :

			Jamè	Raman	Kèk fwa	Souvan
B3	hiv_away	Ou pat lakay ou?	0	1	2	3
B4	hiv_forgot	Ou te bliye?	0	1	2	3
B5	hiv_pills	Te gen twòp grenn pou pran?	0	1	2	3
B6	hiv_se	Ou te pè efè segondè, oswa ou te santi tankou medikaman yo te toksik / danjere?	0	1	2	3
B7	hiv_stigma	Pat vle lòt moun wè ou pran medikaman?	0	1	2	3
B8	hiv_sick	Te santi ou pat byen oswa ou te malad?	0	1	2	3
B9	hiv_depress	Te santi deprime oswa akable?	0	1	2	3
B10	hiv_ranout	Ou pat gen grenn / medikaman ou te fini?	0	1	2	3

B11	hiv_good	Ou te santi w byen?	0	1	2	3
B12	hiv_religion	Kwayans relijyè?	0	1	2	3

Tanpri, ranpli ranpli keksyon pi ba yo apre patisipan an resevwa medikaman ARV li yo

B13	art_refill	*Eske patisipan an te resevwa medikaman ARV li yo?*	☐ 1. Wi<br>☐ 2. Non<br>☐ 3. Li gen medikaman déjà<br><br>*Si non, poukisa?* ______________________________	
B14	art_days	*Li resevwa medikaman pou konbyen jou?*	__________ # jou medikaman	

**SEKSYON C HOSPITALIZATIONS**				
C1	hospitalizations	Èske ou te entène lopital oswa ale nan yon lòt klinik nan mwa ki sot pase a?	☐ 1. Wi→*Refere patisipan an bay yon doktè oswa Miss pou ranpli fòm “**Medical Record Abstraction Form”***<br>☐ 2. Non	

**SEKSYON D: ADERANS ak Medikaman Amlodipin (Hill-Bone Compliance)**				
D1	followup_int	*Eske patisipan an nan bra etid la kap resevwa medikman etid la ki se amlodipin?*	☐ 1. Wi→ ale nan Seksyon B<br>☐ 2. Non→ ale nan fin keksyonè a	
**Koulye a, mwen pral poze kesyon sou nouvo medikaman pou tansyon yo te preskri w ou nan etid la ki rele amlodipine. Nou bezwen konprann ki jan moun ki gen VIH reyèlman ap degaje yo ak grenn yo. Tanpri, di nou kijan sa ye pou ou kounye a. Pa enkyete w pou di nou ke ou pa pran tout grenn ou yo. Nou bezwen konnen kijan sa ye reyèlman, pa di nou sa ou panse nou "vle tande."**				
**Mwen pral poze w keksyon sou medikaman tansyon ki rele amlodidpin lan ke wap pran**				
	Variab	Keksyon	Repons	QC
D2	htn_forget	Èske w janm bliye pran medikaman tansyon an ki rele amlodipin?	☐ 1. Wi<br>☐ 2. Non	
D3	htn_sick	Èske ou konn bliye pran grenn tansyon wo an lè ou santi ou malad?	☐ 1. Wi<br>☐ 2. Non	
D4	htn_better	Èske ou konn bliye pran grenn tansyon wo a lè ou santi ou pi byen?	☐ 1. Wi<br>☐ 2. Non	

EFÈ SEGONDÈ
Kesyon sa yo mande sou sentòm ou ta ka genyen pandan kat semèn ki sot pase yo. Tanpri di mwen, nan lis mwen pral di ou la, kisa ou te santi ki te deranje ou lè wap pran amlodipin lan.

			**Mwen pa gen sentòm sa a**	**Mwen gen sentòm sa epi ...**			
				**Li pa deranje mwen**	**Li deranje m yon ti kras**	**Li deranje m anpil**	**Li deranje mwen anpil anpil**
D5	htn_dizzy	Ou santi tèt ou ap vire oswa ou toudi?	0	1	2	3	4
D6	htn_faint	Ou te endispoze?	0	1	2	3	4
D7	htn_edema	Pye w oswa janm ou te vin anfle?	0	1	2	3	4
D8	htn_other	Nenpòt lòt sentòm, tanpri dekri pou mwen __________________	0	1	2	3	4

Si patisipan an reponn wi nan nenpòt nan youn nan 3 keksyon anwo yo (eskò 1 ou plis), si ou se yon doktè oswa yon enfimyè, tanpri komplete fòm “Adverse Event” ak fòm Egzamen Fisik lan. Si ou se yon psikolog oswa ajan kominotè, tanpri, refere patisipan an nan klinik GHESKIO pou yon doktè oswa yon enfimyè wè li e ranpli fòm yo.

D9	aml_refill	*Eske patisipan te resevwa medikaman amlodipin jodia?*	☐ 1. Wi<br>☐ 2. Non<br>☐ 3. Patisipan an deja gen medikaman amlodipin<br><br>*Si non, poukisa?* ______________________________	
D10	aml_days	*Konbyen jou medikaman amlodipin patisipan an resevwa?*	__________ # jou medikaman amlodipin	
D11	med_other	*Eske yo preskri patisipan lòt medikaman depwi denyè vizit li an?*	☐ 1. Wi<br>☐ 2. Non→FINI	
D12	med_otherwhy	*Si wi, ki medikaman ki te preskri e poukisa?*	______________________________<br>______________________________<br>______________________________	

Remak pou anketè a:
1. Tout sa yo ki EKRI ANGWO LÈT NWA FONSE ta dwe li mo pou mo epi byen fò pou patisipan nan ankèt la.
2. Enstriksyon pou anketè yo ekri an italic epi yo pa dwe li fò.

ENTWODIKSYON KEKSYONÈ A

Bonjou non mwen se _______. Map travay nan Sant GHESKIO kòm _________. Mèsi paske ou dakò patisipe nan ankèt sa. Nou espere enfòmasyon nou kolekte yo ap ede nou konprann tansyon wo kay moun ki enfekte ak VIH pou nou jwenn fason pou nou anpeche ak trete tansyon wo ak maladi kè an Ayiti.

Mwen pral poze ou kèk keksyon pandan apeprè 30 minit sou divès bagay tankou sou sa w fè pou travay, sou zafè lasante ou, tankou si w fimen epi bay kòw aktivite fizik, sou kòman ou santiw, sou kalite lavi ou, ak kominote ou.

Kèk nan keksyon mwen pral poze w yo kapab fè w santi ou pa alèz pou reponn. Ou ka chwazi pa reponn nenpòt keksyon ou pa santi w alèz pou reponn.

Tanpri sonje tout enfòmasyon ou ban nou ap rete sekrè epi yo pap pataje ak nenpòt moun andeyò ekip etid la. Mete w alèz pou w ka reponn keksyon yo avèk onètete jan ou kapab. Si nenpòt nan keksyon sa yo pa klè, tanpri kanpe mwen, epi mwen pral ba w plis eksplikasyon. Sonje byen, pa gen okenn repons ki bon oswa ki pa bon.

Èske w gen yon keksyon anvan nou kòmanse?

Reponn nenpòt enkyetid oswa kesyon anvan ou kontinye.

**SEKSYON C HOSPITALIZASYON**				
C1	hospitalizations	Èske ou te entène lopital oswa ale nan yon lòt klinik nan mwa ki sot pase a?	☐ 1. Wi→*Refere patisipan an bay yon doktè oswa Miss pou ranpli fòm "**Medical Record Abstraction Form**"*<br>☐ 2. Non	

**SEKSYON D: ADERANS ak Medikaman Amlodipin (Hill-Bone Compliance)**				
D1	followup_int	*Eske patisipan an nan bra etid la kap resevwa medikman etid la ki se amlodipin?*	☐ 1. Wi→ ale nan Seksyon B<br>☐ 2. Non→ ale nan fin keksyonè a	
**Koulye a, mwen pral poze kesyon sou nouvo medikaman pou tansyon yo te kòmanse pou ou nan etid la ki rele amlodipine.**<br>**Nou bezwen konprann ki jan moun ki gen VIH reyèlman ap degaje yo ak grenn yo. Tanpri, di nou kijan sa ye pou ou kounye a. Pa enkyete w pou di nou ke ou pa pran tout grenn ou yo. Nou bezwen konnen kijan sa ye reyèlman, pa di nou sa ou panse nou "vle tande."**				
**Mwen pral poze w keksyon sou medikaman tansyon ki rele amlodidpin lan ke wap pran**				
	Variab	Keksyon	Repons	QC
D2	htn_forget	Èske w janm bliye pran medikaman tansyon an ki rele amlodipin?	☐ 1. Wi<br>☐ 2. Non	
D3	htn_sick	Èske ou konn bliye pran grenn tansyon wo an lè ou santi ou malad?	☐ 1. Wi<br>☐ 2. Non	
D4	htn_better	Èske ou konn bliye pran grenn tansyon wo a lè ou santi ou pi byen?	☐ 1. Wi<br>☐ 2. Non	
EFÈ SEGONDÈ<br>**Kesyon sa yo mande sou sentòm ou ta ka genyen pandan kat semèn ki sot pase yo. Tanpri di mwen, nan lis mwen pral di ou la, kisa ou te santi ki te deranje ou lè wap pran amlodipin lan.**				
			**Mwen pa gen sentòm sa a**	**Mwen gen sentòm sa epi ...**

				**Li pa deranje mwen**	**Li deranje m yon ti kras**	**Li deranje m anpil**	**Li deranje mwen anpil anpil**
D5	htn_dizzy	Ou santi tèt ou ap vire oswa ou toudi?	0	1	2	3	4
D6	htn_faint	Ou te endispoze?	0	1	2	3	4
D7	htn_edema	Pye w oswa janm ou te vin anfle?	0	1	2	3	4
D8	htn_other	Nenpòt lòt sentòm, tanpri dekri pou mwen __________________	0	1	2	3	4

Si patisipan an reponn wi nan nenpòt nan youn nan 3 keksyon anwo yo (eskò 1 ou plis), si ou se yon doktè oswa yon enfimyè, tanpri komplete fòm "Adverse Event" ak fòm Egzamen Fisik lan. Si ou se yon psikolog oswa ajan kominotè, tanpri, refere patisipan an nan klinik GHESKIO pou yon doktè oswa yon enfimyè wè li e ranpli fòm yo.

D9	aml_refill	*Eske patisipan te resevwa medikaman amlodipin jodia?*	☐ 1. Wi<br>☐ 2. Non<br>☐ 3. Patisipan an deja gen medikaman amlodipin<br><br>*Si non, poukisa?* ________________________________	
D10	aml_days	*Konbyen jou medikaman amlodipin patisipan an resevwa?*	__________ # jou medikaman amlodipin	
D11	med_other	*Eske yo preskri patisipan lòt medikaman depwi denyè vizit li an?*	☐ 1. Wi<br>☐ 2. Non→FINI	
D12	med_otherwhy	*Si wi, ki medikaman ki te preskri e poukisa?*	________________________________<br>________________________________<br>________________________________	

Remak pou anketè a:
1. Tout sa yo ki EKRI ANGWO LÈT NWA FONSE ta dwe li mo pou mo epi byen fò pou patisipan nan ankèt la.
2. Enstriksyon pou anketè yo ekri an italic epi yo pa dwe li fò.

ENTWODIKSYON ANKÈT LA

Bonjou non mwen se _______. Map travay nan Sant GHESKIO kòm _________. Mèsi paske ou dakò patisipe nan ankèt sa. Sant GHESKIO ap fè yon ankèt sou maladi tansyon kay moun ki enfekte ak VIH. Nou espere enfòmasyon yo kolekte yo ap ede nou konprann tansyon wo kay moun ki enfekte ak VIH pou anpeche ak trete tansyon wo ak maladi kè an Ayiti.

Mwen pral poze w kesyon pou 15 minit sou sijè tankou aderans medikaman, ki jan ou santi ou lè w pran medikaman tansyon yo preskri w la, ak nenpòt ki efè segondè ou ka genyen.

Kèk nan keksyo nmwen pral poze w y okapab fè w santi ou pa alèz pou reponn. Ou ka chwazi pa reponn nenpòt keksyon ou pa santi w alèz pou reponn.

Tanpri sonje tout enfòmasyon ou ban nou ap rete sekrè epi yo pap pataje ak nenpòt moun andeyò ekip etid la. Mete w alèz pou w ka reponn keksyon yo avèk onètete jan ou kapab. Si nenpòt nan keksyon sa yo pa klè, tanpri kanpe mwen, epi mwen pral ba w plis eksplikasyon. Sonje byen, pa gen okenn repons ki bon oswa ki pa bon.

Èske w gen yon keksyon pou mwen anvan nou kòmanse?

Reponn nenpòt enkyetid oswa kesyon anvan ou kontinye.

SESKYON B: ADERANS AK MEDIKAMAN ARV YO (Enstriman mezi ACTG pou aderans, FANMI)
Kounye a mwen pral poze w kèk keksyon sou kijan ou pran medikaman ARV ou yo.

Pifò moun ki gen VIH gen anpil grenn pou yo pran nan diferan moman pandan jounen an.
Anpil moun jwenn li difisil pou yo toujou sonje pran grenn yo:
· Gen kèk moun ki okipe epi bliye pote grenn yo avèk yo.
· Gen kèk moun ki deside sote dòz pou fè pou evite efè segondè oswa jis pa pran grenn jou sa a.

Nou bezwen konprann ki jan moun ki gen VIH reyèlman ap degaje yo ak grenn yo. Tanpri, di nou kijan sa ye pou ou kounye a. Pa enkyete w pou di nou ke ou pa pran tout grenn ou yo. Nou bezwen konnen kijan sa ye reyèlman, pa di nou sa ou panse nou "vle tande."

Kounye a mwen pral poze w kèk keksyon sou kijan ou pran medikaman ARV ou yo.

	Variab	Keksyon	Repons	QC
B1	hiv_medmiss_0M	Nan 4 jou pase yo, konbyen jou ou te pat pran tout dòz medikaman ARV ou yo ?	☐ 0. Okenn<br>☐ 1. 1 jou<br>☐ 2. 2 jou<br>☐ 3. 3 jou<br>☐ 4. 4 jou	
B2	hiv_medwkend_0M	Kèk moun kon di yo konn mal pou sonje pran medikaman yo nan wikenn. Eske ou te bliye pran medikaman ARV ou yo samedi oubyen dimanche ki sot pase a ?	☐ 1. Wi<br>☐ 2. Non	

Pandan mwa ki sot pase a, konbyen fwa ou pa te pran medikaman ARV ou yo pou yon nan rezon sa yo:

			Jamè	Raman	Kèk fwa	Souvan
B1	hiv_away	Ou pat lakay ou?	0	1	2	3
B2	hiv_forgot	Ou te bliye?	0	1	2	3
B3	hiv_pills	Te gen twòp grenn pou pran?	0	1	2	3
B4	hiv_se	Ou te pè efè segondè, oswa ou te santi tankou medikaman yo te toksik / danjere?	0	1	2	3
B5	hiv_stigma	Pat vle lòt moun wè ou pran medikaman?	0	1	2	3
B6	hiv_sick	Te santi ou pat byen oswa ou te malad?	0	1	2	3
B7	hiv_depress	Te santi deprime oswa akable?	0	1	2	3
B8	hiv_ranout	Ou pat gen grenn / medikaman ou te fini?	0	1	2	3
B9	hiv_good	Ou te santi w byen?	0	1	2	3
B10	hiv_religion	Kwayans relijyè?	0	1	2	3

**SEKSYON C: ADERANS ak Medikaman Amlodipin (Hill-Bone Compliance)**				
C1	followup_int	*Eske patisipan an nan bra etid la kap resevwa medikman etid la ki se amlodipin?*	☐ 1. Wi→ ale nan Seksyon C<br>☐ 2. Non→ ale nan Seksyon D	

Koulye a, mwen pral poze kesyon sou nouvo medikaman pou tansyon yo te preskri w ou nan etid la ki rele amlodipine. Nou bezwen konprann ki jan moun ki gen VIH reyèlman ap degaje yo ak grenn yo. Tanpri, di nou kijan sa ye pou ou kounye a. Pa enkyete w pou di nou ke ou pa pran tout grenn ou yo. Nou bezwen konnen kijan sa ye reyèlman, pa di nou sa ou panse nou "vle tande."

Mwen pral poze w keksyon sou medikaman tansyon ki rele amlodidpin lan ke wap pran

	Variab	Keksyon	Repons	QC
C2	htn_forget	Èske w janm bliye pran medikaman tansyon an ki rele amlodipin?	☐ 1. Wi<br>☐ 2. Non	
C3	htn_sick	Èske ou konn bliye pran grenn tansyon wo an lè ou santi ou malad?	☐ 1. Wi<br>☐ 2. Non	
C4	htn_better	Èske ou konn bliye pran grenn tansyon wo a lè ou santi ou pi byen?	☐ 1. Wi<br>☐ 2. Non	

EFÈ SEGONDÈ
Kesyon sa yo mande sou sentòm ou ta ka genyen pandan kat semèn ki sot pase yo. Tanpri di mwen, nan lis mwen pral di ou la, kisa ou te santi ki te deranje ou lè wap pran amlodipin lan.

			**Mwen pa gen sentòm sa a**	**Mwen gen sentòm sa epi ...**			
				**Li pa deranje mwen**	**Li deranje m yon ti kras**	**Li deranje m anpil**	**Li deranje mwen anpil anpil**
C5	htn_dizzy	Ou santi tèt ou ap vire oswa ou toudi?	0	1	2	3	4
C6	htn_faint	Ou te endispoze?	0	1	2	3	4
C7	htn_edema	Pye w oswa janm ou te vin anfle?	0	1	2	3	4
C8	htn_other	Nenpòt lòt sentòm, tanpri dekri pou mwen __________________	0	1	2	3	4

Si patisipan an reponn wi nan nenpòt nan youn nan 3 keksyon anwo yo (eskò 1 ou plis), si ou se yon doktè oswa yon enfimyè, tanpri komplete fòm "Adverse Event" ak fòm Egzamen Fisik lan. Si ou se yon psikolog oswa ajan kominotè, tanpri, refere patisipan an nan klinik GHESKIO pou yon doktè oswa yon enfimyè wè li e ranpli fòm yo.

SEKSYON D: KONPÒTMAN SANTE KI GEN RAPÒ AK MALADI KÈ

ALIMANTASYON: **Kounye a mwen pral poze w keksyon konsènan fwi ak legim ou abitye manje. Mwen pral poze w keksyon tou sou kantite sèl ou sèvi nan manje.**

D1	diet_eatout_12m	Nan yon semèn abityèl, konbyen jou ou manje manje kwit nan men machann, yon restoran oubyen yon kafeterya?	☐ 1. 0-1 jou<br>☐ 2. 2-3 jou<br>☐ 3. 4-5 jou<br>☐ 4. Plis pase 5 jou<br>☐ 99. Pa konnen, refize reponn	
D2	diet_friedfood_12m	Nan yon semèn abityèl, konbyen jou ou manje yon manje ki gen ladan l manje ki fri nan lwil, tankou bannann peze, akra, oubyen griyo?	☐ 1. 0-1 jou<br>☐ 2. 2-3 jou<br>☐ 3. 4-5 jou<br>☐ 4. Plis pase 5 jou<br>☐ 99. Pa konnen, refize reponn	
D3	diet_fruit_serv_12m	Ki kantite fwi ou manje nan yon jou nòmal (ki nan abitid ou)? Lè nou pale de fwi, sa vle di ½ kèp fwi yo oubyen yon fwi ki pa two gwo tankou zaboka oubyen mango. Ji pa ladan l	*Antre yon kantite: # kèp*<br>___________	
D4	diet_veg_serv_12m	Ki kantite legim ou manje nan yon jou nòmal / abityèl? Lè mwen pale de legim	*Antre yon kantite: # kèp*	

		mwen vle di ½ kèp pòmdetè, joumou, oubyen kawòt oubyen 1 kèp fèy legim vèt tankou epina. Mwen pa pale de bannann peze, sòs pwa oubyen diri kole.	___________	
D5	diet salt_use_12m	Konbyen fwa moun ki fè manje oubyen moun ki prepare manje lakay ou itilize sèl kwizin oubyen asezònman ki gen sèl tankou Magi, sèl lay, sèl zonyon, sòy sòs oubyen sòs pwason?	☐ 1.Toujou/Souvan<br>☐ 2. Kèk fwa<br>☐ 3. Raman/Jamè<br>☐ 99. Pa konnen, refize reponn	
D6	diet salt_use2_12m	Konbyen fwa ou ajoute sèl nan manje w oubyen asezònman ki gen sèl tankou Magi, sèl lay, sèl zonyon, sòy sòs, oubyen sòs pwason anvan ou manje l oubyen pandan wap manje l?	☐ 1.Toujou/Souvan<br>☐ 2. Kèk fwa<br>☐ 3. Raman/Jamè<br>☐ 99. Pa konnen, refize reponn	
AKTIVITE FIZIK: **Kounye a, mwen pral poze ou keksyon sou kantite aktivite fizik ou fè, ki gen ladan travay ou ak deyò nan travay ou.**				
D7	activity_type_12m	Eske wap fè travay manyèl? Lè map di travay manyèl, m vle di travay ki fè souf ou wo, fè kè w bat fò pandana plis pase 10 minit. Tankou konstriksyon oubyen agrikiltè.	☐ 1.Yes, Wi<br>☐ 2.No, Non<br>☐ 99. Pa konnen, refize reponn	
D8	activity_moderate_12m	Andeyò travay ou, eske ou fè lòt kalite espò, aktivite fizik oswa aktivite pou pran plezi w ki fè souf ou wo oubyen kè w bat fò (tankou mache vit oubyen netwaye lakay w) pou omwen 150 minit (2 ½ èdtan) nan yon semèn?	☐ 1.Yes, Wi<br>☐ 2.No, Non<br>☐ 99. Pa konnen, refize reponn	
SÈVI AK TABAK **Kounye a mwen ta renmen pozew keksyon sou itilizasyon tabak w.**				
D9	tobacco_ever_12m	Eske ou te janm fimen pou pipiti 100 sigarèt nan tout vi ou?	☐ 1.Yes, Wi<br>☐ 2.No, Non<br>☐ 3. Pa konnen<br>☐ 99. Pa konnen, refize reponn	
D10	tobacco_current_12m	Eske ou fimen nenpòt pwodwi tabak kounye a?	☐ 1. Wi<br>☐ 2. Non=>*Ale ale nan keksyon D12*<br>☐ 99. Pa konnen, refize reponn	
D11	tobacco_daily_12m	Eske ou konn fimen nenpòt pwodwi tabak chak jou kounye a? Lè mwen di chak jou, mwen vle di preske chak jou pou omwen yon lane.	☐ 1.Yes, Wi<br>☐ 2.No, Non<br>☐ 3. Pa konnen<br>☐ 99. Pa konnen, refize reponn	
BWASON KI GEN ALKÒL: **Kounye a mwen ta renmen poze ou keksyon sou itilizasyon alkòl oswa bweson ou.**				
D12	alcohol_currently_12m	Pandan 12 mwa ki sot pase yo, eske ou te bwè alkòl oubyen pwodwi alkòl tankou kleren epi tranpe?	☐ 1. Wi<br>☐ 2. Non=> *fini*<br>☐ 99. Pa konnen, refize reponn	
D13	alcohol_currently_12m	Nan dènye 12 mwa ki sot pase yo, konbyen fwa ou te bwè yon bweson ki gen alkòl? Lè mwen di "bweson ki gen alkòl", mwen vle di yon boutèy 12-ons oubyen yon vè byè, yon vè 5-ons diven, oswa yon bwasson ki gen 1 ti vè likè kòm rhum oubyen kleren.<br>.<br>*Li chwa yo fò pou patisipan an. Chwazi sèlman yon sèl chwa.*	☐ 1. Chak jou<br>☐ 2. 5 - 6 fwa pa semèn<br>☐ 3. 3 - 4 fwa pa semèn<br>☐ 4. 2 fwa pa semèn<br>☐ 5. Yon sèl fwa pa semèn<br>☐ 6. 2 - 3 fwa pa mwa<br>☐ 7. Yon sèl fwa pa mwa<br>☐ 8. 3 – 11 fwa nan lane ki sot pase a<br>☐ 9. 1- 2 fwa nan lane ki sot pase a<br>☐ 99. Pa konnen, refize reponn	
D14	alcohol_quantity_12m	Pandan 12 mwa ki sot pase yo, konbyen bweson ki gen alkòl ou te ka bwè nan jou ou tap bwè alkòl?	*Antre yon chif ant 1-30*<br>____________	

			Si patisipan se yon gason, ale nan keksyon C4.<br>*Si patisipan se yon fi, ale nan keksyon C5.*	
D15	alcohol_max_12m	Pandan 12 mwa ki sot pase yo, konbyen fwa w te bwè 5 oswa plis bweson ki gen nenpòt kalite alkòl nan yon peryòd 2 èdtan? Sa vle di 5 kanèt oubyen boutèy 12-ons byè, 5 vè 5-ons diven oswa 5 bweson ki gen yon sèl ti vè likè tankou rhum oswa kleren.	*Antre yon chif ant 1-30*<br>________	
D16	alcohol_max_12m	Pandan 12 mwa ki sot pase yo, konbyen fwa w te bwè 5 oswa plis bweson ki gen nenpòt kalite alkòl nan yon peryòd 2 èdtan? Sa vle di 5 kanèt oubyen boutèy 12-ons byè, 5 vè 5-ons diven oswa 5 bweson ki gen yon sèl ti vè likè tankou rhum oswa kleren.	*Antre yon chif ant 1-30*<br>________	

Keksyonè a fini la. Mèsi paske w te patisipe nan ankèt sa. Eske ou gen nenpòt keksyon pou mwen?
