# Supplementary material for: Treatment of early hypertension among persons living with HIV in Haiti: Protocol for a randomized controlled trial
Source: PLoS One. 2021 Aug 5;16(8):e0254740. doi: 10.1371/journal.pone.0254740 (PMC8341523; doi:10.1371/journal.pone.0254740)
Supplement: S3 File — (PDF) [file pone.0254740.s004.pdf]

**SECTION A: IDENTIFYING DATA**<br>ENTER ALL OF THE INFORMATION BELOW BEFORE YOU BEGIN THE SURVEY				
	Variable Name	Question	Answer	QC
A1	entry_PID	Study PID:	☐☐☐☐☐☐☐☐	
Enter the time you begin the survey. After you finish the survey, record end time.				
A2	entry_staff	Initials of staff<br>Inisyal Anplwaye a	☐☐	
A3	entry_date	Date of survey:<br>Dat ankèt la	☐☐ ☐☐ ☐☐☐☐<br>DAY MONTH YEAR	
A4	entry_starttime	Time survey start (24hr):<br>Lè ankèt la kòmanse (24è)	☐☐:☐☐	

Notes for Survey Administrator:

1. Scripts that should be read word for word and read out loud to survey participants are in **BOLD.**
2. Instructions to survey administrators are in *Italics* and should not be read out loud.

Remak pou anketè a:

1. Tout sa yo ki **EKRI ANGWO LÈT NWA FONSE** ta dwe li mo pou mo epi byen fò pou patisipan nan ankèt la.
2. Enstriksyon pou *anketè* yo ekri an *italic* epi yo pa dwe li fò.

SURVEY INTRODUCTION

Hello my name is _______. I work at GHESKIO as a __________. Thank you for agreeing to take part. We hope the information collected will help us understand high blood pressure and HIV to prevent and treat hypertension and heart disease in Haiti.

I am going to ask you questions for about 30 minutes on topics such as what you do for work, your health behaviors like smoking and physical activity, how you feel, your quality of life, and about your community.

Some of the questions I ask may be uncomfortable to answer. You can choose not to answer any question that you do not feel comfortable answering.

Please remember that all the information you give us is confidential and will not be shared with anyone outside the study team.

We ask that you answer the questions as honestly as you can.

If any of the questions are unclear, please stop me and I will try and make things clearer.

Remember, there are no right or wrong answers.

Do you have any questions before we begin?
Answer any concerns or questions before proceeding.

ENTWODIKSYON ANKÈT LA

Bonjou non mwen se _______. Map travay nan Sant GHESKIO kòm _________. Mèsi paske ou dakò patisipe nan ankèt sa. Nou espere enfòmasyon yo kolekte yo ap ede nou konprann tansyon wo ak VIH pou anpeche ak trete tansyon wo ak maladi kè an Ayiti.

Mwen pral poze ou kèk keksyon pandan apeprè 30 minit sou divès bagay tankou sou sa w fè pou travay, sou zafè lasante ou, tankou si w fimen epi bay kòw aktivite fizik, sou kòman ou santiw, sou kalite lavi ou, ak kominote ou.

Kèk nan keksyon mwen pral poze w yo kapab fè w santi ou pa alèz pou reponn. Ou ka chwazi pa reponn nenpòt keksyon ou pa santi w alèz pou reponn.

Tanpri sonje tout enfòmasyon ou ban nou ap rete sekrè epi yo pap pataje ak nenpòt moun andeyò ekip etid la.

Mete w alèz pou w ka reponn keksyon yo avèk onètete jan ou kapab.

Si nenpòt nan keksyon sa yo pa klè, tanpri kanpe mwen, epi mwen pral ba w plis eksplikasyon.

Sonje byen, pa gen okenn repons ki bon oswa ki pa bon.

Èske w gen yon keksyon anvan nou kòmanse?

Reponn nenpòt enkyetid oswa kesyon anvan ou kontinye.

**SECTION B: DEMOGRAPHICS AND SOCIOECONOMIC STATUS**<br>**SEKSYON A : ENFÒMASYON SOSYODEMOGRAFIK**<br>**I am going to start by asking you some questions about yourself**<br>**Mwen pral kòmanse poze w kèk keksyon sou ou menm.**				
	Variable Name	Question	Answer	QC
B1	sex	What is your sex?<br>Ki sèks ou?	☐1. Male, Gason<br>☐ 2. Female, Fi<br>☐ 3. Other, lòt<br>☐ 99. Unknown / Refuse to answer, enkoni, refize reponn	
B2	age	What is your age, in years?<br>ki laj ou, nan ane?	__________ years, ane	
B3	ses_education	What is the highest level of school you have attended?<br>Ki pi gwo klas ou fè/rive lekòl?<br><br>*Read options out loud to participant.*<br><br>*Li chwa yo byen fò pou patisipan an*	☐ 1. None, Okenn<br>☐ 2. Primary, Primè<br>☐ 3. Secondary, Segondè<br>☐ 4. Higher, Pi wò pase segondè<br>☐ 99. Unknown / Refuse to answer, enkoni, refize reponn	
B4	ses_marital	What is your current marital status?<br>Eske ou marye (Ki estati sivil ou pou kounye a)?	☐ 1. Single, Selibatè<br>☐ 2. Living together, Plase/Viv avèk<br>☐ 3. Married, Marye<br>☐ 4. Widowed, Vèf/Vèv<br>☐ 5. Divorced/separated, Divòse/Separe<br>☐ 99. Unknown / Refuse to answer, enkoni, refize reponn	
B5	ses_occupation	What best describes your employment status today?<br>Ki travay ou genyen jodia?	☐ 1. Merchant / Own business, Komès<br>☐ 2. Employed, Anplwaye<br>☐ 3. Student, Etidyan	

		If someone is a student and works, mark employed.<br>*Si yon moun ap etidye epi lap travay, tyeke anplwaye.*	☐ 4. Homemaker, Jere kay la<br>☐ 5. Retired, Retrete<br>☐ 6. Not working, pap travay<br>☐ 99. Unknown / Refuse to answer, enkoni, refize reponn	
B6	ses_income	How much income do you earn a day?<br><br>Konbyen lajan yo peye w pa jou?<br><br>*Read options out loud to participant. If participant gives income in gourdes, convert income to Haitian dollars: 1 HD = 5 gourdes.*<br><br>*Li chwa yo byen fò pou patisipan an. Si patisipan an bay lajan li touche a an goud, konvèti li an Dola Ayisyen: 1 HD = 5 goud.*	☐ 1. None, Okenn<br>☐ 2. Less than 10 Haitian dollars a day, Pipiti pase 10 HD/jou<br>☐ 3. 10 to 20 Haitian dollars a day, 11-20 HD/jou<br>☐ 4. 21 to 200 Haitian dollars a day, 21-200 HD/jou<br>☐ 5. More than 200 Haitian dollars a day, Plis pase 200 HD/jou<br>☐ 99. Unknown / Refuse to answer, enkoni, refize reponn	
**MULTIDIMENSIONAL POVETY**<br>**POVETI MULTIDIMENSYONÈL**<br>**Now I'm going to ask you questions about your household and home, to see if you have enough resources to support your health. These questions apply to anyone living in the person's household (relatives, friends, roommates, etc)**<br>**Koulye a, mwen pral poze w kesyon sou kay ou ak lakay ou, pou wè si ou gen ase resous pou sipòte sante ou. Kesyon sa yo aplike a nenpòt moun k ap viv nan kay moun nan (fanmi, zanmi, kolokasyon, elatriye)**				
B7	ses_living_children	How many living children (any age) do you have?<br><br>Konbyen pitit ki vivan ou genyen (kelkeswa laj li/yo)?	_____	
B8	ses_undernourish	In your household, is anyone (adults or children) underweight or undernourished?<br><br>Nan kay ou, èske gen yon moun (granmoun oswa timoun) ki twò piti oswa ki pa gen ase manje?	☐ 1. Yes, Wi<br>☐ 2. No, Non<br>☐ 99. Unknown / Refuse to answer, enkoni, refize reponn	
B9	ses_childdeath	In your household, has any child <18 years died in the last five years?<br><br>Nan kay ou, èske gen timoun <18 ane ki mouri nan senk dènye ane yo?<br><br>*If no children, answer No*	☐ 1. Yes, Wi<br>☐ 2. No, Non<br>☐ 99. Unknown / Refuse to answer, enkoni, refize reponn	
B10	ses_primaryschool	In your household, has anyone completed at least primary school?<br><br>Nan kay ou, èske gen moun ki te konplete omwen lekòl primè?	☐ 1. Yes, Wi<br>☐ 2. No, Non<br>☐ 99. Unknown / Refuse to answer, enkoni, refize reponn	
B11	ses_childschool	In your household, are there any children aged 5-13 years who are not attending school?<br><br>Nan kay ou, èske gen nenpòt timoun ki gen laj 5-13 ane ki pa ale lekòl?	☐ 1. Yes, Wi<br>☐ 2. No, Non<br>☐ 99. Unknown / Refuse to answer, enkoni, refize reponn	
B12	ses_cook	In your household, what type of fuel is used for cooking?<br><br>Nan kay ou, ki kalite gaz ki itilize pou kwit manje?	☐ 1. Dung, wood, charcoal, coal, or grass<br>Dung, bwa, chabon, chabon, oswa zèb<br>☐ 2. Electricity, gas, kerosene<br>Elektrisite, gaz, kewozin	

			☐ 88. Other, lòt<br>☐ 99. Unknown / Refuse to answer, enkoni, refize reponn	
B13	ses_sanitation	In your household, what kind of toilet facility is used?<br><br>Nan kay ou, ki kalite twalèt ki itilize?	☐ 1. Shared latrine, bucket, hanging toilet, bush or field<br>Patrin latrin, bokit, twalèt pandye, ti touf bwa oswa jaden<br>☐ 2. Flush toilet, piped sewer system, flush pit latrine, ventilated improved pit latrine<br>Twalèt kole, sistèm egou kanalize, latrin twou san fon, vantilasyon latrin twou san fon amelyore<br>☐ 88. Other, lòt<br>☐ 99. Unknown / Refuse to answer, enkoni, refize reponn	
B14	ses_water	In your household, what is the main source of drinking water?<br><br>Nan kay ou, ki sous prensipal dlo potab?	☐ 1. Unprotected well, water from spring, surface water (river, stream, lake)<br>Pwoteje byen, dlo ki soti nan sezon prentan, dlo sifas (rivyè, kouran, lak)<br>☐ 2. Piped water, public tapes, boreholes, protected well, rainwater, bottled water<br>Dlo tiyo, kasèt piblik, twou, pwoteje byen, dlo lapli, dlo nan boutèy<br>☐ 88. Other, lòt<br>☐ 99. Unknown / Refuse to answer, enkoni, refize reponn	
B15	ses_electricity	Does your household have electricity?<br><br>Èske kay ou gen elektrisite?	☐ 1. Yes, Wi<br>☐ 2. No, Non<br>☐ 99. Unknown / Refuse to answer, enkoni, refize reponn	
B16	ses_housing	In your household, what is your floor made of?<br><br>Nan kay ou a, ki sa ki etaj ou te fè nan?	☐ 1. Earth, sand, or dung, wood planks<br>Latè, sab, oswa fimye, planch bwa<br>☐ 2. Polished wood, vinyl, ceramic tile, cement, carpet<br>Bwa poli, vinil, mozayik seramik, siman, tapi<br>☐ 99. Unknown / Refuse to answer, enkoni, refize reponn	
B17	ses_items	Does your household have any of the following<br><br>Èske kay ou gen nenpòt nan bagay sa yo<br><br>**Check all that apply**	☐ 1. Radio Radyo<br>☐ 2. Refrigerator Frijidè<br>☐ 3. TV Televizyon<br>☐ 4. Oven/stove Fou / recho<br>☐ 5. Mobile telephone Telefòn mobil<br>☐ 6. Bicycle Bisiklèt<br>☐ 7. Motorbike Motosiklèt<br>☐ 8. Car or Truck Machin oswa kamyon<br>☐ 99. Unknown / Refuse to answer, enkoni, refize reponn	

**SECTION C: CVD HEALTH BEHAVIORS**<br>**SEKSYON C: CVD KOPÒTMAN SANTE**
DIET (WHO STEPS): **Now I am going to ask you questions about the fruits and vegetables you usually eat. I will also ask you questions about your salt consumption.**<br>**Kounye a mwen pral poze w keksyon konsènan fwi ak legim ou abitye manje. Mwen pral poze w keksyon tou sou kantite sèl ou sèvi epi.**

C1	diet_eatout_0M	In a *typical week*, how many days do you eat a meal prepared by a street vendor, a restaurant, or a cafeteria?<br><br>Nan yon semèn konsa a, konbyen jou ou manje manje kwit nan men machann, yon restoran oubyen yon kafeterya?	☐ 1. 0-1 days, 0-1 jou<br>☐ 2. 2-3 days, 2-3 jou<br>☐ 3. 4-5 days, 4-5 jou<br>☐ 4. more than 5 days, Plis ke 5 jou<br>☐ 99. Unknown / Refuse to answer, enkoni, refize reponn	
C2	diet_friedfood_0M	In a *typical week*, how many days do you eat a meal that contains fried foods such as fried plantains, fried tubers or pork?<br><br>Nan yon semèn konsa, konbyen jou ou manje yon manje ki gen ladan l manje ki fri nan lwil, tankou bannann peze, akra, oubyen griyo?	☐ 1. 0-1 days, 0-1 jou<br>☐ 2. 2-3 days, 2-3 jou<br>☐ 3. 4-5 days, 4-5 jou<br>☐ 4. more than 5 days, Plis ke 5 jou<br>☐ 99. Unknown / Refuse to answer, enkoni, refize reponn	
C3	diet_fruit_serv_0M	How many servings of fruit do you eat on a typical day? By fruit, I mean ½ cup or 1 medium fruit such as an avocado or mango. Juice does not count.<br><br>Ki kantite fwi ou manje nan yon jou konsa a? Lè nou pale de fwi, sa vle di ½ kèp fwi yo oubyen yon fwi ki pa two gwo tankou zaboka oubyen mango. Ji pa ladan l	*Enter a number: # servings*<br>*Antre yon kantite: # kèp*<br><br>___________	
C4	diet_veg_serv_0M	How many servings of vegetables do you eat on a typical day? By vegetables, I mean ½ cup of potatoes, pumpkin, or carrots or 1 cup of leafy raw vegetables such as spinach. I do not mean fried plantains, bean sauce or rice.<br><br>Ki kantite legim ou manje nan yon jou konsa a? Lè mwen pale de legim mwen vle di ½ kèp pòmdetè, joumou, oubyen kawòt oubyen 1 kèp fèy legim vèt tankou epina. Mwen pa pale de bannann peze, sòs pwa oubyen diri kole. Ji pa ladan l.	*Enter a number: # servings*<br>*Antre yon kantite: # kèp*<br><br>___________	
C5	diet salt_use_0M	How often is salt or salt-containing seasoning such as Maggi, garlic salt, onion salt, soy sauce or fish sauce used by the person cooking or preparing foods at home?<br><br>Konbyen fwa moun ki fè manje oubyen moun ki prepare manje lakay ou itilize sèl kwizin oubyen asezònman ki gen sèl tankou Magi, sèl lay, sèl zonyon(garlic), sòy sòs oubyen sòs pwason?	☐ 1.Usually/Often, Toujou/Souvan<br>☐ 2.Sometimes, Kèk fwa<br>☐ 3.Rarely/Never, Raman/Jamè<br>☐ 99. Unknown / Refuse to answer, enkoni, refize reponn	
C6	diet salt_use2_0M	How often do you *add* salt or salt-containing seasoning such as Maggi, garlic salt, onion salt, soy sauce or fish sauce to your food before you eat it or when you are eating it?<br><br>Konbyen fwa ou ajoute sèl nan manje w oubyen asezònman ki gen sèl tankou Magi, sèl lay, sèl zonyon (lay), sòy sòs, oubyen sòs pwason anvan ou manje l oubyen pandan wap manje l?	☐ 1.Usually/Often, Toujou/Souvan<br>☐ 2.Sometimes, Kèk fwa<br>☐ 3.Rarely/Never, Raman/Jamè<br>☐ 99. Unknown / Refuse to answer, enkoni, refize reponn	

PHYSICAL ACTIVITY: **Now I am going to ask you questions about the amount of physical activity you do, including your work and outside of your work.**

AKTIVITE FIZIK: **Kounye a, mwen pral poze ou keksyon sou kantite aktivite fizik ou fè, ki gen ladan travay ou ak deyò nan travay ou.**				
C7	activity_type_0M	Do you do manual work related activities? (Manual work is vigorous activity that increases breathing or heart rate for 10 minites or more. Examples include construction or farming.)<br><br>Eske wap fè travay manyèl l? Lè map di travay manyèl, m vle di travay ki fè souf ou wo, fè kè w bat fò plis pase 10 minit. Tankou konstriksyon oubyen agrikiltè.	☐ 1.Yes, Wi<br>☐ 2.No, Non<br>☐ 99. Unknown / Refuse to answer, enkoni, refize reponn	
C8	activity_moderate_0M	Outside of your work activity, do you do any moderate-intensity sports, fitness or recretational activity that causes a small increase in breathing or heart rate (brisk walking or cleaning your house) <u>for at least 150 minutes (2 ½ hours) per week?</u><br><br>Andeyò travay ou, eske ou fè lòt kalite espò, aktivite fizik oswa aktivite pou pran plezi w ki fè souf ou wo oubyen kè w bat fò (tankou mache vit oubyen netwaye lakay w) <u>pou omwen 150 minit (2 ½ èdtan) nan yon semèn</u>?	☐ 1.Yes, Wi<br>☐ 2.No, Non<br>☐ 99. Unknown / Refuse to answer, enkoni, refize reponn	
TOBACCO USE (WHO Steps): **Now I would like to ask you questions about tobacco use.**<br>*SÈVI AK TABAK* **Kounye a mwen ta renmen pozew keksyon sou itilizasyon tabak w.**				
C9	tobacco_ever_0M	Have you ever smoked <u>at least 100 cigarettes</u> in your entire life?<br><br>Eske ou te janm fimen pou pipiti 100 sigarèt nan tout vi ou?	☐ 1.Yes, Wi<br>☐ 2.No, Non<br>☐ 3.I don't know, Pa konnen<br>☐ 99. Unknown / Refuse to answer, enkoni, refize reponn	
C10	tobacco_current_0M	Do you currently smoke any tobacco products?<br><br>Eske ou fimen nenpòt pwodwi tabak kounye a?	☐ 1.Yes, Wi<br>☐ 2.No, Non=>*Skip to question C12*<br>☐ 99. Unknown / Refuse to answer, enkoni, refize reponn	
C11	tobacco_daily_0M	Do you currently smoke any tobacco products daily? By "daily" I mean almost every day for <u>at least one year</u>.<br><br>Eske ou konn fimen nenpòt pwodwi tabak chak jou kounye a? Lè mwen di chak jou, mwen vle di preske chak jou pou omwen yon lane.	☐ 1.Yes, Wi<br>☐ 2.No, Non<br>☐ 99. Unknown / Refuse to answer, enkoni, refize reponn	
ALCOHOL USE (NIDA): **Now I would like to ask you questions about use of alcohol.**<br>*BWASON KI GEN ALKÒL:* **Kounye a mwen ta renmen poze ou keksyon sou itilizasyon alkòl oswa bweson ou.**				
C12	alcohol_currently_0M	During the last 12 months, did you drink alcohol, including alcoholic products such as *tranpe*?<br><br>Pandan 12 mwa ki sot pase yo, eske ou te bwè alkòl oubyen pwodwi alkòl tankou kleren epi tranpe?	☐ 1.Yes, Wi<br>☐ 2.No, Non → *Skip to question D1*<br>☐ 99. Unknown / Refuse to answer , enkoni, refize reponn	

C13	alcohol_currentlyany_0M	During the last 12 months, how often did you usually have any kind of drink containing alcohol? By a drink, I mean a 12.ounce can or glass of beer, a 5.ounce glass of wine, or a drink containing 1 shot of liquor.<br><br>Nan dènye 12 mwa ki sot pase yo, konbyen fwa ou te bwè yon bweson ki gen alkòl? Lè mwen di "bweson ki gen alkòl", mwen vle di yon boutèy 12-ons oubyen yon vè byè, yon vè 5-ons diven, oswa yon bwasson ki gen 1 ti vè likè kòm rhum oubyen kleren.<br><br>*Read answer options to participant. Choose only one option.*<br><br>*Li chwa yo fò pou patisipan an. Chwazi sèlman yon sèl chwa.*	☐ 1. Every day, Chak jou<br>☐ 2. 5 to 6 times a week, 5 - 6 fwa pa semèn<br>☐ 3. 3 to 4 times a week, 3 - 4 fwa pa semèn<br>☐ 4. Twice a week, 2 fwa pa semèn<br>☐ 5. Once a week, Yon sèl fwa pa semèn<br>☐ 6. 2 to 3 times a month, 2 - 3 fwa pa mwa<br>☐ 7. Once a month, Yon sèl fwa pa mwa<br>☐ 8. 3 to 11 times past year, 3 – 11 fwa nan lane ki sot pase a<br>☐ 9. 1 or 2 times past year, 1-2 fwa nan lane ki sot pase a<br>☐ 99. Unknown / Refuse to answer, enkoni, refize reponn	
C14	alcohol_quantity_0M	During the last 12 months, how many alcoholic drinks did you have on a typical day when you drank alcohol?<br><br>Pandan 12 mwa ki sot pase yo, konbyen bweson ki gen alkòl ou te ka bwè nan jou ou tap bwè alkòl?	*Enter a number 1-30*<br>*Antre nimewo antre 1-30*<br><br>____________<br><br>*If participant is male, go to question C4.*<br>*If participant is female, go to question C5.*<br><br>*Si patisipan se yon gason, ale nan keksyon C4.*<br>*Si patisipan se yon fi, ale nan keksyon C5.*	
C15	alcohol_max_0M	During the last 12 months, how often did you have 5 or more drinks containing any kind of alcohol within a *two-hour period*? That would be the equivalent of at least 5 12.ounce cans or bottles of beer, 5 five-ounce glasses of wine or 5 drinks containing one shot of liquor or spirits such as rum or clarin.<br><br>Pandan 12 mwa ki sot pase yo, konbyen fwa w te bwè 5 oswa plis bweson ki gen nenpòt kalite alkòl nan yon peryòd 2 èdtan? Sa vle di 5 kanèt oubyen boutèy 12-ons byè, 5 vè 5-ons diven oswa 5 bweson ki gen yon sèl ti vè likè tankou rhum oswa kleren.	*Enter a number 0-30*<br><br>_________	
C16	alcohol_max_0M	During the last 12 months, how often did you have 4 or more drinks containing any kind of alcohol within a *two-hour period*? That would be the equivalent of at least 4 12.ounce cans or bottles of beer, 4 five-ounce glasses of wine or 4 drinks containing one shot of liquor or spirits such as rum or clarin.<br><br>Pandan 12 mwa ki sot pase yo, konbyen fwa w te bwè 5 oswa plis bweson ki gen	*Enter a number 0-30*<br><br>________	

		nenpòt kalite alkòl nan yon peryòd 2 èdtan? Sa vle di 5 kanèt oubyen boutèy 12-ons byè, 5 vè 5-ons diven oswa 5 bweson ki gen yon sèl ti vè likè tankou rhum oswa kleren.		

SECTION D: MEDICATION ADHERENCE TO ART (ACTG Adherence Instruments, FANMI)
Now I will ask questions on how you take your medications.

Most people with HIV have many pills to take at different times during the day.
Many people find it hard to always remember their pills:

- **Some people get busy and forget to carry their pills with them.**
- **Some people decide to skip doses to avoid side effects or to just not be taking pills that day.**

We need to understand how people with HIV are really doing with their pills. Please tell us what you are actually doing. Don't worry about telling us that you don't take all your pills. We need to know what is really happening, not what you think we "want to hear."

Tanpri poze patisipan yo kesyon sou medikaman.

Pifò moun ki gen VIH gen anpil grenn pou yo pran nan diferan moman pandan jounen an.
Anpil moun jwenn li difisil pou yo toujou sonje grenn yo:
· Gen kèk moun ki okipe epi bliye pote grenn yo avèk yo.
· Gen kèk moun ki deside sote dòz pou fè pou evite efè segondè oswa jis pa pran grenn jou sa a.

Nou bezwen konprann ki jan moun ki gen VIH reyèlman ap fè ak grenn yo. Tanpri, di nou sa ou ye aktyèlman ap fè. Pa enkyete w pou di nou ke ou pa pran tout grenn ou yo. Nou bezwen konnen ki sa ki reyèlman k ap pase, pa sa ou panse nou "vle tande."

Now I will ask you questions about your HIV medications.
Tanpri poze patisipan yo kesyon sou medikaman pou VIH.

	Variable Name	Question	Answer	QC
D1	hiv_medmiss_0M	During the past 4 days, on how many days have you missed taking all your ART doses?<br><br>Nan 4 jou pase yo, konbyen jou ou te bliye pran medikaman ou yo ?	☐ 0. None, Okenn<br>☐ 1. One day, 1 jou<br>☐ 2. Two days, 2 jou<br>☐ 3. Three days, 3 jou<br>☐ 4. Four days, 4 jou	
D2	hiv_medwkend_0M	Some people find that they forget to take their pills on the weekend days. Did you miss any of your anti-HIV medications last weekend— last Saturday or Sunday?<br><br>Kek moun kon di yo bliye pran medikaman yo nan wikenn. Eske ou te bliye medikaman samedi oubyen dimanche ki sot pase a ?	☐ 1. Yes, Wi<br>☐ 2. No, Non	

In the past month, how often have you missed taking your ART medications in the last month because you:

Pandan mwa ki sot pase a, konbyen fwa ou pa te pran medikaman ou yo paske:

			**Never, Jamè**	**Rarely, Raman**	**Sometimes, Kèk fwa**	**Often, Souvan**
D3	hiv_away_0M	Were away from home?<br>Ou te pati lwen lakay ou?	0	1	2	3
D4	hiv_forgot_0M	Simply forgot?<br>Senpleman bliye?	0	1	2	3
D5	hiv_pills_0M	Had too many pills to take?<br>Te gen twòp grenn?	0	1	2	3
D6	hiv_see_0M	Wanted to avoid side effects, or felt like the drug was toxic/harmful?<br>Ou te pè efè segondè, oswa te santi tankou dwòg la te toksik / danjere?	0	1	2	3
D7	hiv_stigma_0M	Did not want others to notice you taking medication?<br>Pa t 'vle lòt moun remake ou pran medikaman?	0	1	2	3
D8	hiv_sick_0M	Felt sick or ill?<br>Te santi malad oswa malad?	0	1	2	3

D9	hiv_depress_0M	Felt depressed/overwhelmed?<br>Te santi deprime oswa akable?	0	1	2	3
D10	hiv_ranout_0M	Ran out of pills?<br>Ou pa te gen grenn?	0	1	2	3
D11	hiv_good_0M	Felt good?<br>Te santi bon?	0	1	2	3
D12	hiv_religion_0M	Religious beliefs?<br>Kwayans relijyon?	0	1	2	3

**This ends the questionnaire. Thank you for taking part in the questionnaire. Do you have any questions?**<br>**Keksyonè anfini la. Mèsi paske w te patisipenan keksyonè an. Eske ou gen keksyonpou mwen?**
☐☐:☐☐<br><br>entry_endtime

Notes for Survey Administrator:

1. Scripts that should be read word for word and read out loud to survey participants are in **BOLD.**
2. Instructions to survey administrators are in *Italics* and should not be read out loud.

Remak pou anketè a:

1. Tout sa yo ki EKRI ANGWO LÈT NWA FONSE ta dwe li mo pou mo epi byen fò pou patisipan nan ankèt la.
2. Enstriksyon pou anketè yo ekri an italic epi yo pa dwe li fò.

SURVEY INTRODUCTION

Hello my name is _______. I work at GHESKIO as a __________. Thank you for agreeing to take part. We hope the information collected will help us understand high blood pressure and HIV to prevent and treat hypertension and heart disease in Haiti.

I am going to ask you questions for 15 minutes on topics such as medication adherence, how you feel about taking the new medication for blood pressure, and any side effects.

Some of the questions I ask may be uncomfortable to answer. You can choose not to answer any question that you do not feel comfortable answering.

Please remember that all the information you give us is confidential and will not be shared with anyone outside the study team.

We ask that you answer the questions as honestly as you can.

If any of the questions are unclear, please stop me and I will try and make things clearer.

Remember, there are no right or wrong answers.

Do you have any questions before we begin?
Answer any concerns or questions before proceeding.

ENTWODIKSYON ANKÈT LA

Bonjou non mwen se _______. Map travay nan Sant GHESKIO kòm _________. Mèsi paske ou dakò patisipenan ankèt sa. Sant GHESKIO ap fè yon ankèt sou maladi tansyon ak VIH. Nou espere enfòmasyon yo kolekte yo ap ede nou konprann tansyon wo ak VIH pou anpeche ak trete tansyon wo ak maladi kè an Ayiti.

Mwen pral poze w kesyon pou 15 minit sou sijè tankou aderans medikaman, ki jan ou santi ou sou pran medikaman an nouvo pou san presyon, ak nenpòt ki efè segondè.

Kèk nan keksyonmwen pral poze w yokapab fè w santiou pa alèz pou reponn. Ou ka chwazi pa reponn nenpòt keksyonou pa santi w alèz pou reponn.

Tanpri sonje tout enfòmasyon ou ban nou ap rete sekrè epi yo pap pataje ak nenpòt moun andeyò ekip etid la.

Mete w alèz pou w kareponn keksyonyo avèk onètete jan ou kapab.

Si nenpòt nan keksyon sa yo pa klè, tanpri kanpe mwen, epi mwenpral ba w plis eksplikasyon.

Sonje byen, pa gen okenn repons ki bon oswa ki pa bon.

Èske w gen yon keksyon anvan nou kòmanse?

Reponn nenpòt enkyetid oswa kesyon anvan ou kontinye.

SECTION B: MEDICATION ADHERENCE TO ART (ACTG Adherence Instruments, FANMI)
Now I will ask questions on how you take your medications.

First I will ask you about your HIV medications.

Most people with HIV have many pills to take at different times during the day.
Many people find it hard to always remember their pills:

- **Some people get busy and forget to carry their pills with them.**
- **Some people decide to skip doses to avoid side effects or to just not be taking pills that day.**

We need to understand how people with HIV are really doing with their pills. Please tell us what you are actually doing. Don't worry about telling us that you don't take all your pills. We need to know what is really happening, not what you think we "want to hear."

Tanpri poze patisipan yo kesyon sou medikaman.

Pifò moun ki gen VIH gen anpil grenn pou yo pran nan diferan moman pandan jounen an.
Anpil moun jwenn li difisil pou yo toujou sonje grenn yo:
· Gen kèk moun ki okipe epi bliye pote grenn yo avèk yo.
· Gen kèk moun ki deside sote dòz pou fè pou evite efè segondè oswa jis pa pran grenn jou sa a.

Nou bezwen konprann ki jan moun ki gen VIH reyèlman ap fè ak grenn yo. Tanpri, di nou sa ou ye aktyèlman ap fè. Pa enkyete w pou di nou ke ou pa pran tout grenn ou yo. Nou bezwen konnen ki sa ki reyèlman k ap pase, pa sa ou panse nou "vle tande."

**Now I will ask you questions about your HIV medications.**<br>**Tanpri poze patisipan yo kesyon sou medikaman pou VIH.**				
	Variable Name	Question	Answer	QC
B1	hiv_medmiss	During the past 4 days, on how many days have you missed taking all your ART doses?<br><br>Nan 4 jou pase yo, konbyen jou ou te bliye pran medikaman ou yo ?	☐ 0. None, Okenn<br>☐ 1. One day, 1 jou<br>☐ 2. Two days, 2 jou<br>☐ 3. Three days, 3 jou<br>☐ 4. Four days, 4 jou	
B2	hiv_medwkend	Some people find that they forget to take their pills on the weekend days. Did you miss any of your anti-HIV medications last weekend— last Saturday or Sunday?<br><br>Kek moun kon di yo bliye pran medikaman yo nan wikenn. Eske ou te bliye medikaman samedi oubyen dimanche ki sot pase a ?	☐ 1. Yes, Wi<br>☐ 2. No, Non	

In the past month, how often have you missed taking your ART medications in the last month because you:

Pandan mwa ki sot pase a, konbyen fwa ou pa te pran medikaman ou yo paske:

			**Never, Jamè**	**Rarely, Raman**	**Sometimes, Kèk fwa**	**Often, Souvan**
B3	hiv_away	Were away from home?<br>Ou te pati lwen lakay ou?	0	1	2	3
B4	hiv_forgot	Simply forgot?<br>Senpleman bliye?	0	1	2	3
B5	hiv_pills	Had too many pills to take?<br>Te gen twòp grenn?	0	1	2	3

B6	hiv_se	Wanted to avoid side effects, or felt like the drug was toxic/harmful?<br>Ou te pè efè segondè, oswa te santi tankou dwòg la te toksik / danjere?	0	1	2	3
B7	hiv_stigma	Did not want others to notice you taking medication?<br>Pa t 'vle lòt moun remake ou pran medikaman?	0	1	2	3
B8	hiv_sick	Felt sick or ill?<br>Te santi malad oswa malad?	0	1	2	3
B9	hiv_depress	Felt depressed/overwhelmed?<br>Te santi deprime oswa akable?	0	1	2	3
B10	hiv_ranout	Ran out of pills?<br>Ou pa te gen grenn?	0	1	2	3
B11	hiv_good	Felt good?<br>Te santi bon?	0	1	2	3
B12	hiv_religion	Religious beliefs?<br>Kwayans relijyon?	0	1	2	3

Staff member, please fill out the below after giving patient their ART medications

B13	art_refill	*Was the patient's ART refill given?*	☐ 1. Yes<br>☐ 2. No<br>☐ 3. Patient already has medications<br><br>*If not, why?* ______________________	
B14	art_days	*Number of days of ART given*	__________ days of medications	

SECTION C HOSPITALIZATIONS				
C1	hospitalizations	Have you been hospitalized or gone to another clinic in the past month?<br><br>Èske ou te entène lopital oswa ale nan yon lòt klinik nan mwa ki sot pase a?	☐ 1. Yes, Wi→*Refer patient to MD or RN to fill out* ***Medical Record Abstraction Form***<br>☐ 2. No, Non	

**SECTION D: MEDICATION ADHERENCE to Amlodipine (Hill-Bone Compliance)**				
D1	followup_int	*Is the participant in the intervention arm (receiving amlodipine as a study drug?)*	☐ 1. Yes→ proceed to Section B<br>☐ 2. No→ End Survey	

Now I will ask questions on the new blood pressure medication started as part of this study, amlodipine.

Again, we need to understand how people with HIV are really doing with their pills. Please tell us what you are actually doing. Don't worry about telling us that you don't take all your pills. We need to know what is really happening, not what you think we "want to hear."

Koulye a, mwen pral poze kesyon sou nouvo medikaman san presyon an te kòmanse kòm yon pati nan etid sa a, amlodipine.

Nou bezwen konprann ki jan moun ki gen VIH reyèlman ap fè ak grenn yo. Tanpri, di nou sa ou ye aktyèlman ap fè. Pa enkyete w pou di nou ke ou pa pran tout grenn ou yo. Nou bezwen konnen ki sa ki reyèlman k ap pase, pa sa ou panse nou "vle tande."

**Now I will ask you questions about the blood pressure medication amlodipine.**<br>**Tanpri poze patisipan yo kesyon sou medikaman amlodipine.**				
	Variable Name	Question	Answer	QC
D2	htn_forget	Do you ever forget to take your high blood pressure medication amlodipine?<br>Èske w janm bliye pran medikaman tansyon wo ou amlodipin?	☐ 1. Yes, Wi<br>☐ 2. No, Non	
D3	htn_sick	Do you ever miss taking your high blood pressure pills when you feel sick?<br>Èske ou janm manke pran grenn tansyon wo ou lè ou santi ou malad?	☐ 1. Yes, Wi<br>☐ 2. No, Non	
D4	htn_better	Do you ever miss taking your high blood pressure pills when you feel better?<br>Èske ou janm manke pran grenn tansyon ou lè ou santi ou pi byen?	☐ 1. Yes, Wi<br>☐ 2. No, Non	

SIDE EFFECTS
EFÈ KOTE
The following questions ask about symptoms you might have had during the past four weeks. Please check the box that describes how much you have been bothered by each symptom from amlodipine.

Kesyon sa yo mande sou sentòm ou ta ka genyen pandan kat semèn ki sot pase yo. Tanpri tcheke ti bwat la ki dekri konbyen ou te anmande pa chak sentòm soti nan amlodipin.

			**I do not have this symptom** **Mwen pa gen sentòm sa a**	**I have this symptom and…** **Mwen gen sentòm sa a ak ...**			
				**It doesn't bother me** **Li pa deranje mwen**	**It bothers me a little** **Li deranje m 'yon ti kras**	**It bothers me a lot** **Li deranje m 'anpil**	**It bothers me terribly** **Li deranje mwen fò anpil**
D5	htn_dizzy	Feeling dizzy or lightheaded?<br>Ou santi ou tèt vire oswa toudi?	0	1	2	3	4
D6	htn_faint	Fainted?	0	1	2	3	4

		Endispoze?					
D7	htn_edema	Have new swelling in your feet or legs?<br>Fè nouvo anfle nan pye ou oswa janm ou?	0	1	2	3	4
D8	htn_other	Any other symptom, please list ____________<br>Nenpòt lòt sentòm, tanpri lisv____________	0	1	2	3	4

If patient answers yes to any of the above 3 questions (score of 1 or higher), if you are an RN or MD, please complete an Adverse Event Form, and a Physical Exam. If you are a Community Health Worker, please refer the patient to the GHESKIO Clinic to have an RN or MD fill out an Adverse Event Form, and a Physical Exam.

D9	aml_refill	*Was the patient's amlodipine refill given?*	☐ 1. Yes<br>☐ 2. No<br>☐ 3. Patient already has medications<br><br>*If not, why?* ________________________	
D10	aml_days	*Number of days of amlodipine given*	__________ days of medications	
D11	med_other	*Has any other medication been started on the patient since the last visit?*	☐ 1. Yes<br>☐ 2. No→END	
D12	med_otherwhy	*If Yes, what medication has been started and why?*	____________________<br>____________________<br>____________________	

Notes for Survey Administrator:

1. Scripts that should be read word for word and read out loud to survey participants are in **BOLD.**
2. Instructions to survey administrators are in *Italics* and should not be read out loud.

Remak pou anketè a:

1. Tout sa yo ki EKRI ANGWO LÈT NWA FONSE ta dwe li mo pou mo epi byen fò pou patisipan nan ankèt la.
2. Enstriksyon pou anketè yo ekri an italic epi yo pa dwe li fò.

SURVEY INTRODUCTION

Hello my name is _______. I work at GHESKIO as a __________. Thank you for agreeing to take part. We hope the information collected will help us understand high blood pressure and HIV to prevent and treat hypertension and heart disease in Haiti.

I am going to ask you questions for 15 minutes on topics such as medication adherence, how you feel about taking the new medication for blood pressure, and any side effects.

Some of the questions I ask may be uncomfortable to answer. You can choose not to answer any question that you do not feel comfortable answering.

Please remember that all the information you give us is confidential and will not be shared with anyone outside the study team.

We ask that you answer the questions as honestly as you can.

If any of the questions are unclear, please stop me and I will try and make things clearer.

Remember, there are no right or wrong answers.

Do you have any questions before we begin?
Answer any concerns or questions before proceeding.

ENTWODIKSYON ANKÈT LA

Bonjou non mwen se _______. Map travay nan Sant GHESKIO kòm _________. Mèsi paske ou dakò patisipenan ankèt sa. Sant GHESKIO ap fè yon ankèt sou maladi tansyon ak VIH. Nou espere enfòmasyon yo kolekte yo ap ede nou konprann tansyon wo ak VIH pou anpeche ak trete tansyon wo ak maladi kè an Ayiti.

Mwen pral poze w kesyon pou 15 minit sou sijè tankou aderans medikaman, ki jan ou santi ou sou pran medikaman an nouvo pou san presyon, ak nenpòt ki efè segondè.

Kèk nan keksyonmwen pral poze w yokapab fè w santiou pa alèz pou reponn. Ou ka chwazi pa reponn nenpòt keksyonou pa santi w alèz pou reponn.

Tanpri sonje tout enfòmasyon ou ban nou ap rete sekrè epi yo pap pataje ak nenpòt moun andeyò ekip etid la.

Mete w alèz pou w kareponn keksyonyo avèk onètete jan ou kapab.

Si nenpòt nan keksyon sa yo pa klè, tanpri kanpe mwen, epi mwenpral ba w plis eksplikasyon.

Sonje byen, pa gen okenn repons ki bon oswa ki pa bon.

Èske w gen yon keksyon anvan nou kòmanse?

Reponn nenpòt enkyetid oswa kesyon anvan ou kontinye.

**SECTION C HOSPITALIZATIONS**				
C1	hospitalizations	Have you been hospitalized or gone to another clinic in the past month?<br><br>Èske ou te entène lopital oswa ale nan yon lòt klinik nan mwa ki sot pase a?	☐ 1. Yes, Wi→*Refer patient to MD or RN to fill out* ***Medical Record Abstraction Form***<br>☐ 2. No, Non	

**SECTION D: MEDICATION ADHERENCE to Amlodipine (Hill-Bone Compliance)**				
D1	followup_int	*Is the participant in the intervention arm (receiving amlodipine as a study drug?)*	☐ 1. Yes→ proceed to Section B<br>☐ 2. No→ End Survey	

Now I will ask questions on the new blood pressure medication started as part of this study, amlodipine.

Again, we need to understand how people with HIV are really doing with their pills. Please tell us what you are actually doing. Don't worry about telling us that you don't take all your pills. We need to know what is really happening, not what you think we "want to hear."

Koulye a, mwen pral poze kesyon sou nouvo medikaman san presyon an te kòmanse kòm yon pati nan etid sa a, amlodipine.

Nou bezwen konprann ki jan moun ki gen VIH reyèlman ap fè ak grenn yo. Tanpri, di nou sa ou ye aktyèlman ap fè. Pa enkyete w pou di nou ke ou pa pran tout grenn ou yo. Nou bezwen konnen ki sa ki reyèlman k ap pase, pa sa ou panse nou "vle tande."

Now I will ask you questions about the blood pressure medication amlodipine.
Tanpri poze patisipan yo kesyon sou medikaman amlodipine.

	Variable Name	Question	Answer	QC
D2	htn_forget	Do you ever forget to take your high blood pressure medication amlodipine?<br>Èske w janm bliye pran medikaman tansyon wo ou amlodipin?	☐ 1. Yes, Wi<br>☐ 2. No, Non	
D3	htn_sick	Do you ever miss taking your high blood pressure pills when you feel sick?<br>Èske ou janm manke pran grenn tansyon wo ou lè ou santi ou malad?	☐ 1. Yes, Wi<br>☐ 2. No, Non	
D4	htn_better	Do you ever miss taking your high blood pressure pills when you feel better?<br>Èske ou janm manke pran grenn tansyon ou lè ou santi ou pi byen?	☐ 1. Yes, Wi<br>☐ 2. No, Non	

SIDE EFFECTS
EFÈ KOTE
The following questions ask about symptoms you might have had during the past four weeks. Please check the box that describes how much you have been bothered by each symptom from amlodipine.

Kesyon sa yo mande sou sentòm ou ta ka genyen pandan kat semèn ki sot pase yo. Tanpri tcheke ti bwat la ki dekri konbyen ou te anmande pa chak sentòm soti nan amlodipin.

			**I do not have this symptom** **Mwen pa gen**	**I have this symptom and…**<br><br>**Mwen gen sentòm sa a ak ...**

			**sentòm sa a**				
				**It doesn't bother me** **Li pa deranje mwen**	**It bothers me a little** **Li deranje m 'yon ti kras**	**It bothers me a lot** **Li deranje m 'anpil**	**It bothers me terribly** **Li deranje mwen fò anpil**
D5	htn_dizzy	Feeling dizzy or lightheaded?<br>Ou santi ou tèt vire oswa toudi?	0	1	2	3	4
D6	htn_faint	Fainted?<br>Endispoze?	0	1	2	3	4
D7	htn_edema	Have new swelling in your feet or legs?<br>Fè nouvo anfle nan pye ou oswa janm ou?	0	1	2	3	4
D8	htn_other	Any other symptom, please list ____________<br>Nenpòt lòt sentòm, tanpri lisv____________	0	1	2	3	4

If patient answers yes to any of the above 3 questions (score of 1 or higher), if you are an RN or MD, please complete an Adverse Event Form, and a Physical Exam. If you are a Community Health Worker, please refer the patient to the GHESKIO Clinic to have an RN or MD fill out an Adverse Event Form, and a Physical Exam.

D9	aml_refill	*Was the patient's amlodipine refill given?*	☐ 1. Yes<br>☐ 2. No<br>☐ 3. Patient already has medications<br><br>*If not, why?* ________________________________	
D10	aml_days	*Number of days of amlodipine given*	__________ days of medications	
D11	med_other	*Has any other medication been started on the patient since the last visit?*	☐ 1. Yes<br>☐ 2. No→END	
D12	med_otherwhy	*If Yes, what medication has been started and why?*	________________________________<br>________________________________<br>________________________________	

Notes for Survey Administrator:

1. Scripts that should be read word for word and read out loud to survey participants are in **BOLD.**
2. Instructions to survey administrators are in *Italics* and should not be read out loud.

Remak pou anketè a:

1. Tout sa yo ki EKRI ANGWO LÈT NWA FONSE ta dwe li mo pou mo epi byen fò pou patisipan nan ankèt la.
2. Enstriksyon pou anketè yo ekri an italic epi yo pa dwe li fò.

SURVEY INTRODUCTION

Hello my name is _______. I work at GHESKIO as a __________. Thank you for agreeing to take part. We hope the information collected will help us understand high blood pressure and HIV to prevent and treat hypertension and heart disease in Haiti.

I am going to ask you questions for 15 minutes on topics such as medication adherence, how you feel about taking the new medication for blood pressure, and any side effects.

Some of the questions I ask may be uncomfortable to answer. You can choose not to answer any question that you do not feel comfortable answering.

Please remember that all the information you give us is confidential and will not be shared with anyone outside the study team.

We ask that you answer the questions as honestly as you can.

If any of the questions are unclear, please stop me and I will try and make things clearer.

Remember, there are no right or wrong answers.

Do you have any questions before we begin?
Answer any concerns or questions before proceeding.

ENTWODIKSYON ANKÈT LA

Bonjou non mwen se _______. Map travay nan Sant GHESKIO kòm _________. Mèsi paske ou dakò patisipenan ankèt sa. Sant GHESKIO ap fè yon ankèt sou maladi tansyon ak VIH. Nou espere enfòmasyon yo kolekte yo ap ede nou konprann tansyon wo ak VIH pou anpeche ak trete tansyon wo ak maladi kè an Ayiti.

Mwen pral poze w kesyon pou 15 minit sou sijè tankou aderans medikaman, ki jan ou santi ou sou pran medikaman an nouvo pou san presyon, ak nenpòt ki efè segondè.

Kèk nan keksyonmwen pral poze w yokapab fè w santiou pa alèz pou reponn. Ou ka chwazi pa reponn nenpòt keksyonou pa santi w alèz pou reponn.

Tanpri sonje tout enfòmasyon ou ban nou ap rete sekrè epi yo pap pataje ak nenpòt moun andeyò ekip etid la.

Mete w alèz pou w kareponn keksyonyo avèk onètete jan ou kapab.

Si nenpòt nan keksyon sa yo pa klè, tanpri kanpe mwen, epi mwenpral ba w plis eksplikasyon.

Sonje byen, pa gen okenn repons ki bon oswa ki pa bon.

Èske w gen yon keksyon anvan nou kòmanse?

Reponn nenpòt enkyetid oswa kesyon anvan ou kontinye.

SECTION B: MEDICATION ADHERENCE TO ART (ACTG Adherence Instruments, FANMI)
Now I will ask questions on how you take your medications.

First I will ask you about your HIV medications.

Most people with HIV have many pills to take at different times during the day.
Many people find it hard to always remember their pills:

- **Some people get busy and forget to carry their pills with them.**
- **Some people decide to skip doses to avoid side effects or to just not be taking pills that day.**

We need to understand how people with HIV are really doing with their pills. Please tell us what you are actually doing. Don't worry about telling us that you don't take all your pills. We need to know what is really happening, not what you think we "want to hear."

Tanpri poze patisipan yo kesyon sou medikaman.

Pifò moun ki gen VIH gen anpil grenn pou yo pran nan diferan moman pandan jounen an.
Anpil moun jwenn li difisil pou yo toujou sonje grenn yo:
· Gen kèk moun ki okipe epi bliye pote grenn yo avèk yo.
· Gen kèk moun ki deside sote dòz pou fè pou evite efè segondè oswa jis pa pran grenn jou sa a.

Nou bezwen konprann ki jan moun ki gen VIH reyèlman ap fè ak grenn yo. Tanpri, di nou sa ou ye aktyèlman ap fè. Pa enkyete w pou di nou ke ou pa pran tout grenn ou yo. Nou bezwen konnen ki sa ki reyèlman k ap pase, pa sa ou panse nou "vle tande."

Now I will ask you questions about your HIV medications.
Tanpri poze patisipan yo kesyon sou medikaman pou VIH.

	Variable Name	Question	Answer	QC
B1	hiv_medmiss_12M	During the past 4 days, on how many days have you missed taking all your ART doses?<br><br>Nan 4 jou pase yo, konbyen jou ou te bliye pran medikaman ou yo ?	☐ 0. None, Okenn<br>☐ 1. One day, 1 jou<br>☐ 2. Two days, 2 jou<br>☐ 3. Three days, 3 jou<br>☐ 4. Four days, 4 jou	
B2	hiv_medwkend_12M	Some people find that they forget to take their pills on the weekend days. Did you miss any of your anti-HIV medications last weekend— last Saturday or Sunday?<br><br>Kek moun kon di yo bliye pran medikaman yo nan wikenn. Eske ou te bliye medikaman samedi oubyen dimanche ki sot pase a ?	☐ 1. Yes, Wi<br>☐ 2. No, Non	

In the past month, how often have you missed taking your ART medications in the last month because you:

Pandan mwa ki sot pase a, konbyen fwa ou pa te pran medikaman ou yo paske:

			Never, Jamè	Rarely, Raman	Sometimes, Kèk fwa	Often, Souvan
B1	hiv_away	Were away from home?<br>Ou te pati lwen lakay ou?	0	1	2	3
B2	hiv_forgot	Simply forgot?<br>Senpleman bliye?	0	1	2	3
B3	hiv_pills	Had too many pills to take?<br>Te gen twòp grenn?	0	1	2	3
B4	hiv_se	Wanted to avoid side effects, or felt like the drug was toxic/harmful?	0	1	2	3

		Ou te pè efè segondè, oswa te santi tankou dwòg la te toksik / danjere?				
B5	hiv_stigma	Did not want others to notice you taking medication?<br>Pa t 'vle lòt moun remake ou pran medikaman?	0	1	2	3
B6	hiv_sick	Felt sick or ill?<br>Te santi malad oswa malad?	0	1	2	3
B7	hiv_depress	Felt depressed/overwhelmed?<br>Te santi deprime oswa akable?	0	1	2	3
B8	hiv_ranout	Ran out of pills?<br>Ou pa te gen grenn?	0	1	2	3
B9	hiv_good	Felt good?<br>Te santi bon?	0	1	2	3
B10	hiv_religion	Religious beliefs?<br>Kwayans relijyon?	0	1	2	3

**SECTION C: MEDICATION ADHERENCE to Amlodipine (Hill-Bone Compliance)**

C1	followup_int	*Is the participant in the intervention arm (receiving amlodipine as a study drug?)*	☐ 1. Yes→ proceed to Section B<br>☐ 2. No→ End Survey	

Now I will ask questions on the new blood pressure medication started as part of this study, amlodipine.

Again, we need to understand how people with HIV are really doing with their pills. Please tell us what you are actually doing. Don't worry about telling us that you don't take all your pills. We need to know what is really happening, not what you think we "want to hear."

Koulye a, mwen pral poze kesyon sou nouvo medikaman san presyon an te kòmanse kòm yon pati nan etid sa a, amlodipine.

Nou bezwen konprann ki jan moun ki gen VIH reyèlman ap fè ak grenn yo. Tanpri, di nou sa ou ye aktyèlman ap fè. Pa enkyete w pou di nou ke ou pa pran tout grenn ou yo. Nou bezwen konnen ki sa ki reyèlman k ap pase, pa sa ou panse nou "vle tande."

Now I will ask you questions about the blood pressure medication amlodipine.
Tanpri poze patisipan yo kesyon sou medikaman amlodipine.

	Variable Name	Question	Answer	QC
C2	htn_forget	Do you ever forget to take your high blood pressure medication amlodipine?<br>Èske w janm bliye pran medikaman tansyon wo ou amlodipin?	☐ 1. Yes, Wi<br>☐ 2. No, Non	
C3	htn_sick	Do you ever miss taking your high blood pressure pills when you feel sick?<br>Èske ou janm manke pran grenn tansyon wo ou lè ou santi ou malad?	☐ 1. Yes, Wi<br>☐ 2. No, Non	
C4	htn_better	Do you ever miss taking your high blood pressure pills when you feel better?<br>Èske ou janm manke pran grenn tansyon ou lè ou santi ou pi byen?	☐ 1. Yes, Wi<br>☐ 2. No, Non	

SIDE EFFECTS
EFÈ KOTE
The following questions ask about symptoms you might have had during the past four weeks. Please check the box that describes how much you have been bothered by each symptom from amlodipine.

Kesyon sa yo mande sou sentòm ou ta ka genyen pandan kat semèn ki sot pase yo. Tanpri tcheke ti bwat la ki dekri konbyen ou te anmande pa chak sentòm soti nan amlodipin.

			**I do not have this symptom** **Mwen pa gen sentòm sa a**	**I have this symptom and…** **Mwen gen sentòm sa a ak ...**			
				**It doesn't bother me** **Li pa deranje mwen**	**It bothers me a little** **Li deranje m 'yon ti kras**	**It bothers me a lot** **Li deranje m 'anpil**	**It bothers me terribly** **Li deranje mwen fò anpil**
C5	htn_dizzy	Feeling dizzy or lightheaded?	0	1	2	3	4

		Ou santi ou tèt vire oswa toudi?					
C6	htn_faint	Fainted?<br>Endispoze?	0	1	2	3	4
C7	htn_edema	Have new swelling in your feet or legs?<br>Fè nouvo anfle nan pye ou oswa janm ou?	0	1	2	3	4
C8	htn_other	Any other symptom, please list _____________<br>Nenpòt lòt sentòm, tanpri lisv_____________	0	1	2	3	4

If patient answers yes to any of the above 3 questions (score of 1 or higher), if you are an RN or MD, please complete an Adverse Event Form, and a Physical Exam. If you are not an RN or MD, please refer the patient to an RN or MD to complete the required forms.

**SECTION D: CVD HEALTH BEHAVIORS**<br>**SEKSYON D: CVD KOPÒTMAN SANTE**				
DIET (WHO STEPS): **Now I am going to ask you questions about the fruits and vegetables you usually eat. I will also ask you questions about your salt consumption and the type of oil you use for cooking.**<br>**Kounye a mwen pral poze w keksyon konsènan fwi ak legim ou abitye manje. Mwen pral poze w keksyon tou sou kantite sèl ou sèvi epi.**				
D1	diet_eatout_12m	In a *typical week*, how many days do you eat a meal prepared by a street vendor, a restaurant, or a cafeteria?<br><br>Nan yon semèn konsa a, konbyen jou ou manje manje kwit nan men machann, yon restoran oubyen yon kafeterya?	☐ 1. 0-1 days, 0-1 jou<br>☐ 2. 2-3 days, 2-3 jou<br>☐ 3. 4-5 days, 4-5 jou<br>☐ 4. more than 5 days, Plis ke 5 jou<br>☐ 99. Unknown / Refuse to answer, enkoni, refize reponn	
D2	diet_friedfood_12m	In a *typical week*, how many days do you eat a meal that contains fried foods such as fried plantains, fried tubers or pork?<br><br>Nan yon semèn konsa, konbyen jou ou manje yon manje ki gen ladan l manje ki fri nan lwil, tankou bannann peze, akra, oubyen griyo?	☐ 1. 0-1 days, 0-1 jou<br>☐ 2. 2-3 days, 2-3 jou<br>☐ 3. 4-5 days, 4-5 jou<br>☐ 4. more than 5 days, Plis ke 5 jou<br>☐ 99. Unknown / Refuse to answer, enkoni, refize reponn	
D3	diet_fruit_serv_12m	How many servings of fruit do you eat on a typical day? By fruit, I mean ½ cup or 1 medium fruit such as an avocado or mango. Juice does not count.<br><br>Ki kantite fwi ou manje nan yon jou konsa a? Lè nou pale de fwi, sa vle di ½ kèp fwi yo oubyen yon fwi ki pa two gwo tankou zaboka oubyen mango. Ji pa ladan l	*Enter a number: # servings*<br>*Antre yon kantite: # kèp*<br><br>___________	
D4	diet_veg_serv_12m	How many servings of vegetables do you eat on a typical day? By vegetables, I mean ½ cup of potatoes, pumpkin, or carrots or 1 cup of leafy raw vegetables such as spinach. I do not mean fried plantains, bean sauce or rice.<br><br>Ki kantite legim ou manje nan yon jou konsa a? Lè mwen pale de legim mwen vle di ½ kèp pòmdetè, joumou, oubyen kawòt oubyen 1 kèp fèy legim vèt tankou epina. Mwen pa pale de bannann peze, sòs pwa oubyen diri kole. Ji pa ladan l.	*Enter a number: # servings*<br>*Antre yon kantite: # kèp*<br><br>___________	
D5	diet salt_use_12m	How often is salt or salt-containing seasoning such as Maggi, garlic salt, onion salt, soy sauce or fish sauce used by the person cooking or preparing foods at home?<br><br>Konbyen fwa moun ki fè manje oubyen moun ki prepare manje lakay ou itilize sèl kwizin oubyen asezònman ki gen sèl tankou Magi, sèl lay, sèl zonyon(garlic), sòy sòs oubyen sòs pwason?	☐ 1.Usually/Often, Toujou/Souvan<br>☐ 2.Sometimes, Kèk fwa<br>☐ 3.Rarely/Never, Raman/Jamè<br>☐ 99. Unknown / Refuse to answer, enkoni, refize reponn	
D6	diet salt_use2_12m	How often do you *add* salt or salt-containing seasoning such as Maggi, garlic salt, onion salt, soy sauce or fish sauce to your food before you eat it or when you are eating it?<br><br>Konbyen fwa ou ajoute sèl nan manje w oubyen asezònman ki gen sèl tankou Magi,	☐ 1.Usually/Often, Toujou/Souvan<br>☐ 2.Sometimes, Kèk fwa<br>☐ 3.Rarely/Never, Raman/Jamè<br>☐ 99. Unknown / Refuse to answer, enkoni, refize reponn	

		sèl lay, sèl zonyon (lay), sòy sòs, oubyen sòs pwason anvan ou manje l oubyen pandan wap manje l?		
PHYSICAL ACTIVITY: **Now I am going to ask you questions about the amount of physical activity you do, including your work and outside of your work.**<br>*AKTIVITE FIZIK*: **Kounye a, mwen pral poze ou keksyon sou kantite aktivite fizik ou fè, ki gen ladan travay ou ak deyò nan travay ou.**				
D7	activity_type_12m	Do you do manual work related activities? (Manual work is vigorous activity that increases breathing or heart rate for 10 minites or more. Examples include construction or farming.)<br><br>Eske wap fè travay manyèl l? Lè map di travay manyèl, m vle di travay ki fè souf ou wo, fè kè w bat fò plis pase 10 minit. Tankou konstriksyon oubyen agrikiltè.	☐ 1.Yes, Wi<br>☐ 2.No, Non<br>☐ 99. Unknown / Refuse to answer, enkoni, refize reponn	
D8	activity_moderate_12m	Outside of your work activity, do you do any moderate-intensity sports, fitness or recretational activity that causes a small increase in breathing or heart rate (brisk walking or cleaning your house) <u>for at least 150 minutes (2 ½ hours) per week?</u><br><br>Andeyò travay ou, eske ou fè lòt kalite espò, aktivite fizik oswa aktivite pou pran plezi w ki fè souf ou wo oubyen kè w bat fò (tankou mache vit oubyen netwaye lakay w) <u>pou omwen 150 minit (2 ½ èdtan) nan yon semèn</u>?	☐ 1.Yes, Wi<br>☐ 2.No, Non<br>☐ 99. Unknown / Refuse to answer, enkoni, refize reponn	
TOBACCO USE (WHO Steps): **Now I would like to ask you questions about tobacco use.**				
D9	tobacco_ever_12m	Have you ever smoked <u>at least 100 cigarettes</u> in your entire life?<br><br>Eske ou te janm fimen pou pipiti 100 sigarèt nan tout vi ou?	☐ 1.Yes, Wi<br>☐ 2.No, Non<br>☐ 3.I don't know, Pa konnen<br>☐ 99. Unknown / Refuse to answer, enkoni, refize reponn	
D10	tobacco_current_12m	Do you currently smoke any tobacco products?<br><br>Eske ou fimen nenpòt pwodwi tabak kounye a?	☐ 1.Yes, Wi<br>☐ 2.No, Non=>*Skip to question C12*<br>☐ 99. Unknown / Refuse to answer, enkoni, refize reponn	
D11	tobacco_daily_12m	Do you currently smoke any tobacco products daily? By "daily" I mean almost every day for <u>at least one year</u>.<br><br>Eske ou konn fimen nenpòt pwodwi tabak chak jou kounye a? Lè mwen di chak jou, mwen vle di preske chak jou pou omwen yon lane.	☐ 1.Yes, Wi<br>☐ 2.No, Non<br>☐ 99. Unknown / Refuse to answer, enkoni, refize reponn	
ALCOHOL USE (NIDA): **Now I would like to ask you questions about use of alcohol.**				
D12	alcohol_currently_12m	During the last 12 months, did you drink alcohol, including alcoholic products such as *tranpe*?<br><br>Pandan 12 mwa ki sot pase yo, eske ou te bwè alkòl oubyen pwodwi alkòl tankou kleren epi tranpe?	☐ 1.Yes, Wi<br>☐ 2.No, Non → *Skip to question D1*<br>☐ 99. Unknown / Refuse to answer , enkoni, refize reponn	

D13	alcohol_currently_12m	During the last 12 months, how often did you usually have any kind of drink containing alcohol? By a drink, I mean a 12.ounce can or glass of beer, a 5.ounce glass of wine, or a drink containing 1 shot of liquor.<br><br>Nan dènye 12 mwa ki sot pase yo, konbyen fwa ou te bwè yon bweson ki gen alkòl? Lè mwen di "bweson ki gen alkòl", mwen vle di yon boutèy 12-ons oubyen yon vè byè, yon vè 5-ons diven, oswa yon bwasson ki gen 1 ti vè likè kòm rhum oubyen kleren.<br><br>*Read answer options to participant. Choose only one option.*<br><br>*Li chwa yo fò pou patisipan an. Chwazi sèlman yon sèl chwa.*	☐ 1. Every day, Chak jou<br>☐ 2. 5 to 6 times a week, 5 - 6 fwa pa semèn<br>☐ 3. 3 to 4 times a week, 3 - 4 fwa pa semèn<br>☐ 4. Twice a week, 2 fwa pa semèn<br>☐ 5. Once a week, Yon sèl fwa pa semèn<br>☐ 6. 2 to 3 times a month, 2 - 3 fwa pa mwa<br>☐ 7. Once a month, Yon sèl fwa pa mwa<br>☐ 8. 3 to 11 times past year, 3 – 11 fwa nan lane ki sot pase a<br>☐ 9. 1 or 2 times past year, 1-2 fwa nan lane ki sot pase a<br>☐ 99. Unknown / Refuse to answer, enkoni, refize reponn	
D14	alcohol_quantity_12m	During the last 12 months, how many alcoholic drinks did you have on a typical day when you drank alcohol?<br><br>Pandan 12 mwa ki sot pase yo, konbyen bweson ki gen alkòl ou te ka bwè nan jou ou tap bwè alkòl?	*Enter a number 1-30*<br>*Antre nimewo antre 1-30*<br><br>____________<br><br>*If participant is male, go to question C4.*<br>*If participant is female, go to question C5.*<br><br>*Si patisipan se yon gason, ale nan keksyon C4.*<br>*Si patisipan se yon fi, ale nan keksyon C5.*	
D15	alcohol_max_12m	During the last 12 months, how often did you have 5 or more drinks containing any kind of alcohol within a *two-hour period*? That would be the equivalent of at least 5 12.ounce cans or bottles of beer, 5 five-ounce glasses of wine or 5 drinks containing one shot of liquor or spirits such as rum or clarin.<br><br>Pandan 12 mwa ki sot pase yo, konbyen fwa w te bwè 5 oswa plis bweson ki gen nenpòt kalite alkòl nan yon peryòd 2 èdtan? Sa vle di 5 kanèt oubyen boutèy 12-ons byè, 5 vè 5-ons diven oswa 5 bweson ki gen yon sèl ti vè likè tankou rhum oswa kleren.	*Enter a number 0-30*<br><br>_________	
D16	alcohol_max_12m	During the last 12 months, how often did you have 4 or more drinks containing any kind of alcohol within a *two-hour period*? That would be the equivalent of at least 4 12.ounce cans or bottles of beer, 4 five-ounce glasses of wine or 4 drinks containing one shot of liquor or spirits such as rum or clarin.	*Enter a number 0-30*<br><br>________	

		Pandan 12 mwa ki sot pase yo, konbyen fwa w te bwè 5 oswa plis bweson ki gen nenpòt kalite alkòl nan yon peryòd 2 èdtan? Sa vle di 5 kanèt oubyen boutèy 12-ons byè, 5 vè 5-ons diven oswa 5 bweson ki gen yon sèl ti vè likè tankou rhum oswa kleren.		

This ends the questionnaire. Thank you for taking part in the questionnaire. Do you have any questions?
Keksyonè anfini la. Mèsi paske w te patisipenan keksyonè an. Eske ou gen keksyonpou mwen?
